# Synthesis and In Silico Docking Study towards M-Pro of Novel Heterocyclic Compounds Derived from Pyrazolopyrimidinone as Putative SARS-CoV-2 Inhibitors

**DOI:** 10.3390/molecules27165303

**Published:** 2022-08-19

**Authors:** Mabrouk Horchani, Niels V. Heise, René Csuk, Hichem Ben Jannet, Abdel Halim Harrath, Anis Romdhane

**Affiliations:** 1 Laboratory of Heterocyclic Chemistry, Natural Products and Reactivity, Medicinal Chemistry and Natural Products (LR11ES39), Faculty of Sciences of Monastir, University of Monastir, Monastir 5000, Tunisia; 2 Department of Organic Chemistry, Martin-Luther-University Halle-Wittenberg, Kurt-Mothes-Str. 2, D-06120 Halle, Germany; 3 Department of Zoology, College of Science, King Saud University, Riyad 11451, Saudi Arabia

**Keywords:** pyrazolopyrimidinone, diketone, cyclic anhydride, pyrazole, 2,5-pyrrolidinedione, docking, ADMET, COVID-19, Omicron

## Abstract

In addition to vaccines, antiviral drugs are essential in order to suppress COVID-19. Although some inhibitor candidates have been determined to target the SARS-CoV-2 protein, there is still an urgent need to continue researching novel inhibitors of the SARS-CoV-2 main protease ‘Omicron P132H’, a protein that has recently been discovered. In the present study, in the search for therapeutic alternatives to treat COVID-19 and its recent variants, we conducted a structure-based virtual screening using docking studies for a new series of pyrazolo[3,4-*d*]pyrimidin-4(5*H*)-one derivatives **5**–**13**, which were synthesized from the condensation reaction of pyrazolopyrimidinone-hydrazide (**4**) with a series of electrophiles. Some significant ADMET predictions–in addition to the docking results–were obtained based on the types of interactions formed and the binding energy values were compared to the reference anti- SARS-CoV-2 redocked drug nirmatrelvir.

## 1. Introduction

At the end of 2019, a new coronavirus named SARS-CoV-2 appeared in Wuhan, China, and caused an unusual outbreak of viral pneumonia. Highly contagious, this infection was caused by the novel coronavirus, also known as coronavirus disease 2019 (COVID-19) [1]. According to the World Health Organization (WHO), about 14.9 million excess deaths were associated with the COVID-19 pandemic in 2020 and 2021 [2]. The main clinical symptoms of this virus are fever, fatigue, headache, dry cough, and runny nose. These problems are specifically associated with difficulties in breathing, which often lead to the patient’s death. The WHO declared this pandemic to be one of the most dangerous health catastrophes in human history [3] as this virus accelerated faster than experts predicted [4].

Nowadays, many variants of SARS-CoV-2 are appearing; these variants hold altered spike proteins, hence allowing the virus to nullify the effectiveness of the vaccines. The Centers for Disease Control and Prevention (CDC) has classified SARS-CoV-2 variants as viruses of concern, interest, and high importance. Thus, several variants of SARS-CoV-2 have been identified and deemed a source of long-term risk of infection in immunocompromised individuals [5]. The SARS-CoV-2 main protease (M^pro^) is a cysteine protease that is critical for the treatment of the two polyproteins (pp1a and pp1ab) encoded by the SARS-CoV-2 genome. M^pro^ plays an indispensable role in the replication cycle of the coronavirus. In more detail, the M^pro^ operates at more than 11 cleavage sites on the pp1ab [6]. The recognition sequence is Leu-Gln for most of the 11 sites [7]. Inhibiting the role of M^pro^ would essentially block any viral replication [8]. There are no known homologs of M^pro^ in humans with an identical cleavage specificity. Thereby, its inhibition is unlikely to show any side effects, making it an important target for COVID-19 drugs.

On 26 November 2021, the World Health Organization’s Technical Advisory Group on Virus Evolution (TAG-VE) proposed that the variant B.1.1.529, commonly known as Omicron, was associated with a substantial ability to evade immunity from prior infections [9]. The TAG-VE made this decision after finding that the Omicron variant has several mutations that might influence how quickly it spreads or the danger of the disease it causes. Further, the Omicron variant is considered as the most divergent strain seen in large numbers so far during the COVID-19 pandemic, raising concerns that it may be related to greater transmissibility, lower vaccine efficacy, and an increased risk of reinfection [10,11,12,13]. As known, the whole world is experiencing a difficult time at many economic and social levels because of COVID-19.Therefore, many studies have been carried out in order to end this pandemic [14,15,16].

Unfortunately over time, SARS-CoV-2, which is the viral strain behind the ongoing global pandemic of COVID-19, is still considered a global health issue [17,18] and its menace keeps growing with the emergence of many newly evolved strains [19,20,21,22]. The highly contagious nature of this life-threatening virus makes it imperative to find therapies to counteract its spread. It is known that vaccinations are considered a preventive measure, but the search for effective treatments remains of paramount importance [23,24,25].

For a long time, nitrogen heterocycles attracted the interest of both synthetic and biological researchers because of their various chemical, biological, and pharmacological properties [26,27]. Many of them, in particular those incorporating a pyrimidine scaffold fused with one or more heterocyclic fragments, exhibited a broad range of biological activities such as cytotoxic [28], anti-microbial [29], anti-tyrosinase [30], and anti-cholinesterase activities [31].

**Figure 1:**
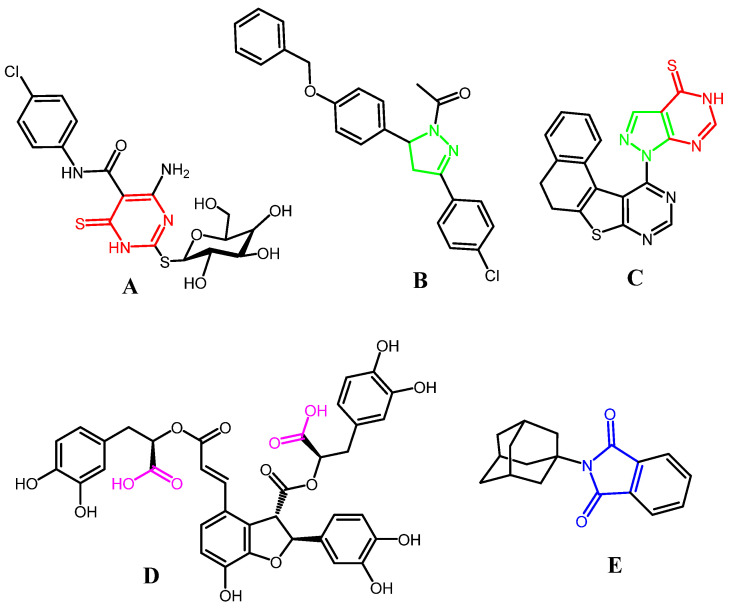
Previously reported antiviral compounds (**A**–**E**).

Furthermore, several synthetic molecules containing heterocyclic moieties are currently known for their antiviral potentials, such as pyrimidinone [32] (Figure 1A), pyrazole [33] (Figure 1B), pyrazole fused pyrimidinone [34] (Figure 1C), acid: COVID-19-inhibitor candidate [35] (Figure 1D), and the 2,5-pyrrolidinedione [36] (Figure 1E) systems.

Encouraged by these findings, in this study, we have designed and synthesized a new series of heterocyclic compounds via the combination of a pyrazolopyrimidinone moiety with different pharmacophores, such as pyrazole, 2,5-pyrrolidinedione, and the carboxylic acid function. Furthermore, using a combined two-step track of virtual screening and molecular docking methods, we have screened our collection of synthesized compounds. Thereby, molecules holding a high structural diversity were selected in order to cover a broad range of chemical spaces in the enzyme pockets. So, the virtual screening experiments were performed by applying the blind docking mode of the AutoDock Vina software in order to predict the inhibition of the target proteins corresponding to Omicron. Furthermore, chemoinformatic tools were used to examine the ADMET properties, pharmacokinetic parameters, and toxicological characteristics of the synthesized analogues.

## 2. Results and Discussion

### 2.1. Chemistry

The justification of a large part of the chemistry directed towards the access to new compounds containing nitrogen at the fusion of the rings, is due to the exploitation of numerous molecules that have interesting biological potentials, such as antiviral potentials, in the field of medicinal chemistry.

**Scheme 1:**
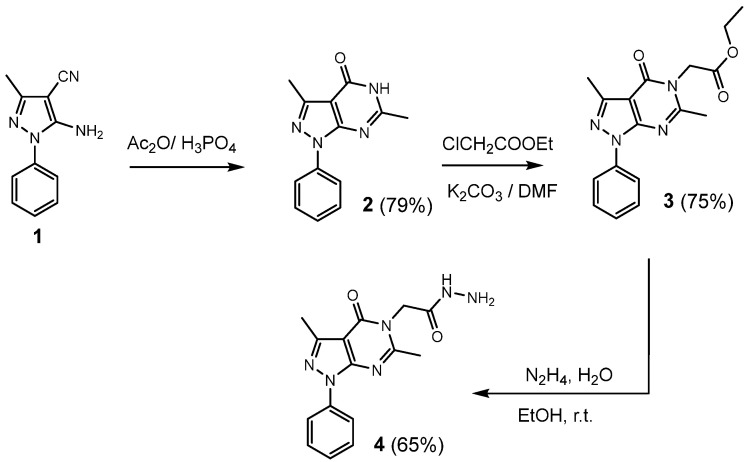
Synthetic pathway to the precursors **2**–**4**.

In order to access our target molecules **5**–**13**, we started by preparing 5-amino-3-methyl-1-phenyl-1*H*-pyrazole-4-carbonitrile (**1**), in accordance with our previously reported method [28]. The hydrazides constitute an essential intermediate for the synthesis of several heterocyclic compounds [37,38,39] and are known for their high reactivity in addition to their utility as building blocks. This stirred our interest for their use in the preparation of the desired compounds **5**–**13**. Our approach to the target molecules started with the synthesis of the key intermediate (**4**). Herein, we are reporting a scalable methodology for the synthesis of hydrazide (**4**) (65% yield). The synthetic route is illustrated in Scheme 1. The reaction sequence began with the preparation of pyrazolopyrimidinone (**2**), by heating a solution of the precursor **1** in an acetic anhydride in the presence of phosphoric acid under reflux. The reaction of the compound (**2**) with ethyl chloroacetate gave the corresponding ester (**3**). The latter was then submitted to a nucleophilic substitution with hydrazine monohydrate affording the 2-(3,6-dimethyl-4-oxo-1-phenyl-1,4-dihydro-5*H*-pyrazolo[3,4-*d*]pyrimidin-5-yl)acetohydrazide (**4**). The structures of compounds **1**–**4** were established on the basis of their ^1^H and ^13^C-NMR and MS spectra, as well as their elemental analysis data after purification; their complete spectral data are given in the experimental section.

**Scheme 2:**
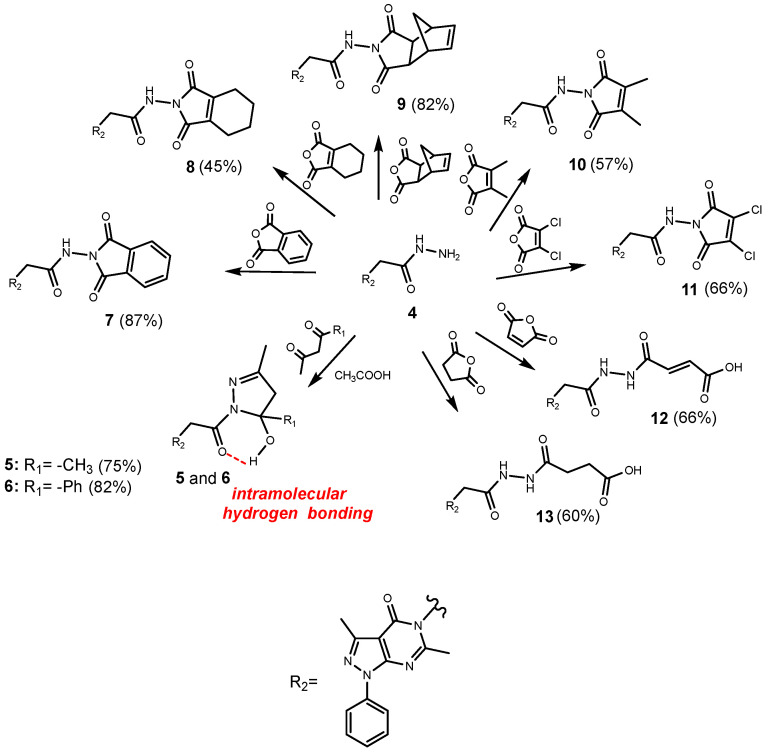
Synthetic pathway to the compounds **5**–**13**.

The condensation of the precursor **4** with pentane-2,4-dione or 1-phenyl-1,3-butanedione in the presence of a catalytic amount of acetic acid in refluxing dioxane was carried out for 5 h. This afforded the new hydroxylated pyrazole derivatives **5** and **6**, respectively, via the Knorr pyrazole synthesis mechanism [30]. Furthermore, the hydrazide **4** was reacted with a series of cyclic anhydrides in refluxing 1,4-dioxane, in the presence of a catalytic amount of acetic acid over a period of7–9 h in order to yield the compounds **7**–**13** (Scheme 2).

The structures of the target compounds were characterized by ^1^H and ^13^C-NMR and MS spectra, as well as by their microanalytical data. Indeed, the ^1^H-NMR spectra of the compounds **5**–**13** showed the disappearance of the signals at δ_H_ 4.45 corresponding to the –NH_2_ function of the hydrazide **4** and the appearance of new signals assigned to the hydrogen introduced by the different electrophilic species used. In addition, the ^1^H NMRanalysis wasenriched by some 2D experiments. For example, in the case of the compound **5**, the Knorr-pyrazole’s structure was evidenced with the help of the HMBC spectrum that exhibited the correlations between H-20/C-18, H-20/C-17, and H-19/C-17. This sequence is consolidated by COSY and NOESY experiments that show the homonuclear correlations between H-20/H-17 and H-19/H-17. The analysis of the ^13^C-NMR spectra also confirmed the structure of the compounds. In addition, the ESI-MS spectra of the examined compounds **5**–**13** showed the correct pseudo-molecular ions [M − H]^−^; in addition, the elemental analysis data were consistent with the molecular formulas.

### 2.2. Molecular Docking Studies

#### 2.2.1. Insight into the Protein Used

**Figure 2:**
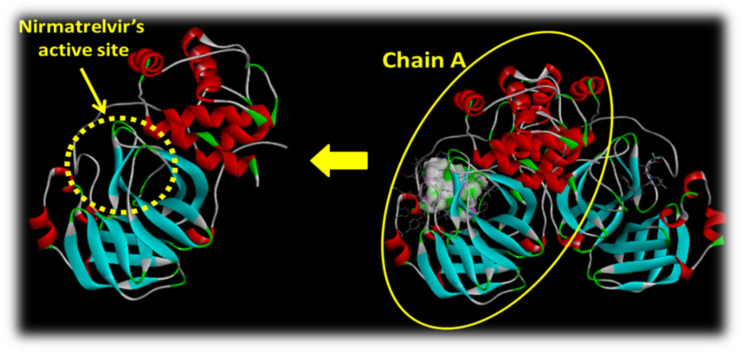
(**Right**): the structure of SARS-CoV-2 M^pro^ Omicron P132H in complex with nirmatrelvir. (**Left**): chain A of main protease enzyme.

Thanks to the efforts of many structural biology and crystallography research groups, we actually have access to a significant number of SARS-CoV-2-related protein structures (spike protein, papain-like protease, and main protease) in the Protein Data Bank. Based on the importance of M-pro detailed in the introduction and which is considered as an optimal target for the search of inhibitors potentially usable as drugs in the curing of SARS-CoV-2 infections, the SARS-CoVs-2: Omicron (pdb:7tll, chain A) was considered and selected as a good COVID-19 target for this study (Figure 2). Our goal was to determine the modes of interaction of the protein-ligand complexes, thereby looking for favorable orientations for the binding of the ligand to the receptor in the active site of nirmatrelvir.

#### 2.2.2. Discussion of the Different Interactions Formed

**Figure 3:**
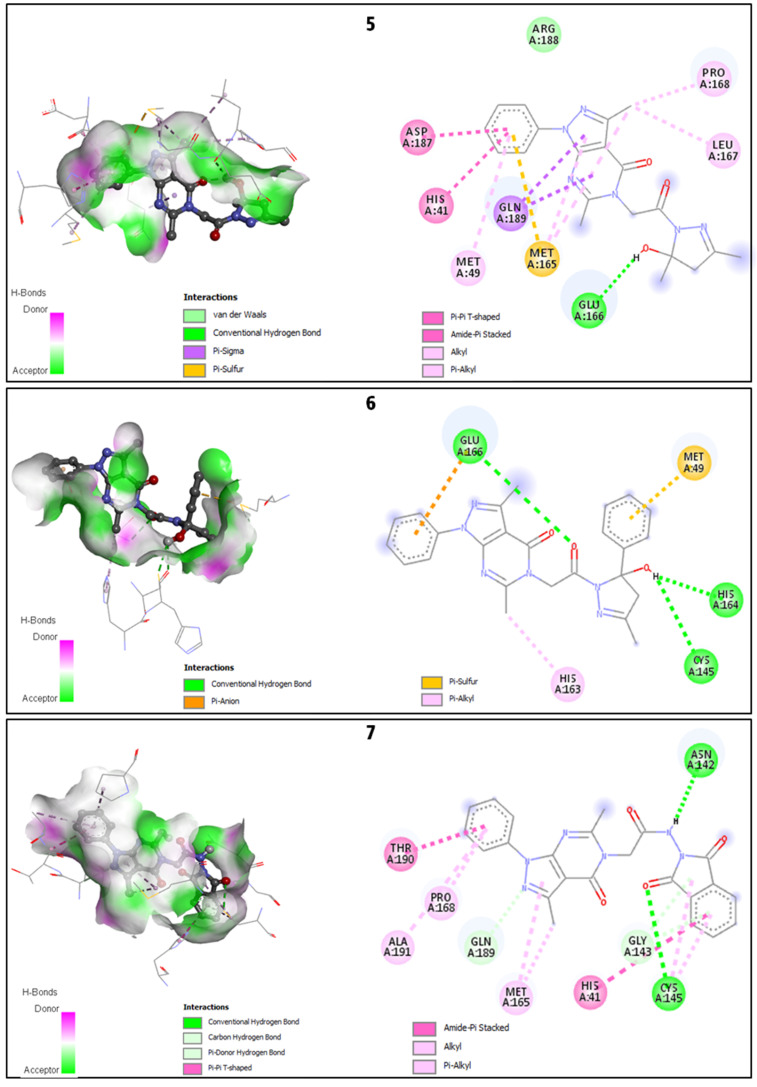
Representation of the 3D structure of the docked compounds **5**, **6,** and **7** bound to the pocket region of SARS-CoV-2 M^pro^ with the hydrogen bond (**left**) and the corresponding 2D diagram of the interactions (**right**).

**Figure 4:**
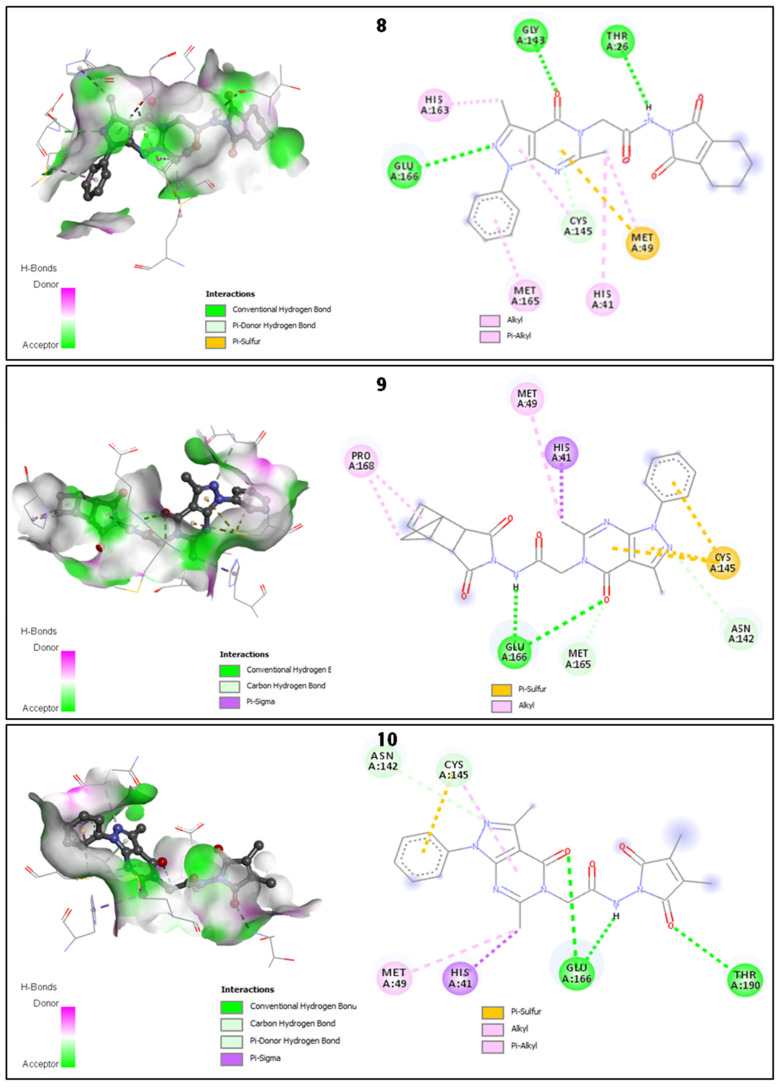
Representation of the 3D structure of the docked compounds **8**, **9,** and **10** bound to the pocket region of SARS-CoV-2 M^pro^ with the hydrogen bond (**left**) and the corresponding 2D diagram of the interactions (**right**).

**Figure 5:**
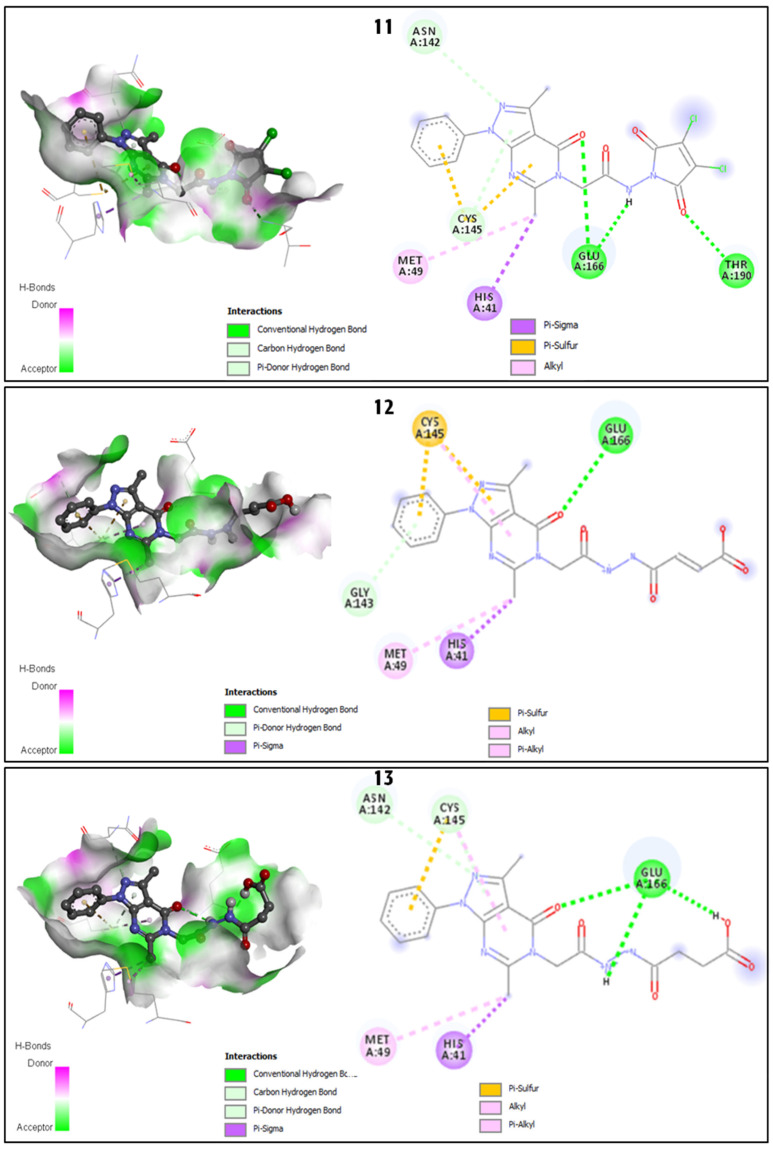
Representation of the 3D structure of the docked compounds **11**, **12,** and **13** bound to the pocket region of SARS-CoV-2 M^pro^ with the hydrogen bond (**left**) and the corresponding 2D diagram of the interactions (**right**).

**Figure 6:**
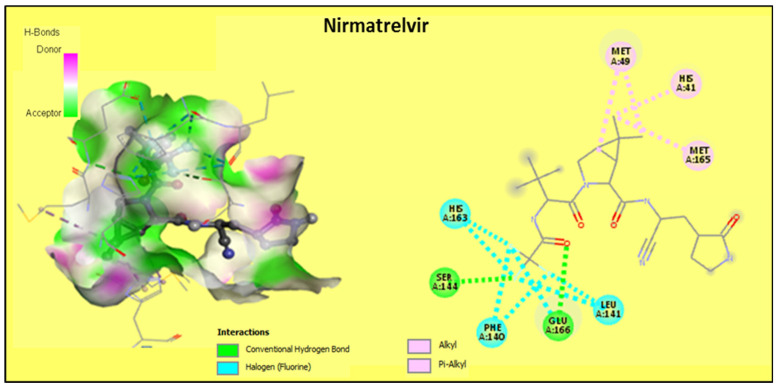
Representation of the 3D structure of the docked nirmatrelvir bound to the pocket region of SARS-CoV-2 M^pro^ with the hydrogen bond (**left**) and the corresponding 2D diagram of the interactions (**right**).

The molecular docking studies of the synthesized compounds **5**–**13**, towards the ‘SARS-CoV-2 M^pro^ Omicron P132H’ receptor were investigated and the obtained results are summarized in Table 1. It is essential to mention that all of the docked molecules against the target enzyme COVID-19 are ranked according to their binding energy, and a quick check of each molecule’s total interactions with nrmatrelvir binding site was performed effectively by counting the total number of hydrogen bonds. Figure 3, Figure 4, Figure 5 and Figure 6 revealed that the docked ligands are involved in many interesting hydrogen bonds and hydrophobic interactions which demonstrate a good protein inhibition of some synthesized molecules. Indeed, as a detailed description of all docked compounds, it can be noted that the compound **5** which contains, in its structure, a hydroxylated pyrazole fragment that formed a conventional H-bond with GLU166 via the hydroxyl group. Furthermore, its importance as an inhibitor is perceptible through many hydrophobic interactions such as: Pi-Sigma with GLN189, Pi-Sulfur with MET165, Pi-Pi T-shaped with HIS41, Amide-Pi stacked with ASP187, Pi-Alkyl with MET49, MET165 and Alkyl with MET165, LEU167, and PRO168 via the phenylpyrazolopyrimidinone moiety, while the analogue **6** engages mainly by the three H-bonds displayed with residues: CYS145, HIS164, and GLU166. Furthermore, the compounds **7**–**11** that consist of pyrazolopyrimidine linked to 2,5-pyrrolidinediones, showed interesting docking results by at least two hydrogen bonds by the NH and CO groups; we cite the case of the compound **7**, which showed H-bonds with ASN142, CYS145, and likewise the compound **9** with GLU166, in addition to other hydrophobic interactions. The compounds **8**, **10,** and **11** each presented three H-bonds in addition to several other interactions. For more detail, the compound **8** formed, through its phenylpyrazolopyrimidinone pharmacophore, a Pi-Sulfur with MET49, Pi-Alkyl with CYS145 and MET165, and Alkyl interactions with HIS41, MET49, and HIS163. Furthermore, compounds **10** and **11** showed the same types of interactions: three H-bonds via the NH and CO groups with amino acids: GLU166 and THR190, Pi-Sigma with HIS41, alkyl with MET49, Pi-Sulfur with CYS145, carbon hydrogen bond with CYS145, and the Pi-donor hydrogen bond with ASN142. Regarding the acid derivatives, the compound **12** displayed a single H-bond with GLU166 in addition to other hydrophobic bonds similar to those achieved by the compounds **10** and **11**, while **13** is involved in three conventional hydrogen bonds with the residue GLU166. Some of the above results are more significant than those given by the reference drug nirmatrelvir which only formed two H bonds with SER144 and GLU166. The present in silico results showed an interesting anti-M^pro^ potential for most of our synthesized pyrazolopyrimidinone derivatives (binding affinity= −7.5 to −8.2 kcal/mol) compared to the redocked nirmatrelvir (binding affinity= −7.7 kcal/mol), and also to previous works showing similar results with the repurposed drugs darunavir (−7.4 kcal/mol), lopinavir (−7.3 kcal/mol), as well as to some polyphenol compounds (binding energy = −7.2 to −8.2 kcal/mol) [40]. This comparison is supported by some data from the same study and is based on the importance of the H-bonds for the inhibition of M^pro^, that showed that some of our compounds showed 3 hydrogen bonds with the residues Glu166, Gly143, and sometimes also with Cys145, while darunavir showed only two (with Glu166, Gly143) and lopinavir showed only one with Cys145.

#### 2.2.3. Binding Energies Analysis

The nature and number of the interactions exerted by the synthesized compounds with the different amino acids are not on their own sufficient in order to predict their anti-SARS CoV-2 potency. It is also very useful to discuss the value of the energy of the complex (compound-protein) resulting from these interactions: the lower the energy, the more stable the complex and hence, the better the activity. The compound **8** that holds a 4,5,6,7-hexahydro-2*H*-isoindol-2-yl)acetamide fragment, exhibited the lowest energy value (8.2 kcal/mol) followed by its analogue **7** (R = phthalic) with an energy value of 8.2 kcal/mol. Then, the compound **13** with its acid function, displayed an energy value of 7.8 kcal/mol. These findings suggest that these three derivatives may have the most significant activity against the Omicron variant of SARS-CoV-2 compared to the rest of the analogues and the reference drug nirmatrelvir (7.7 kcal/mol) (Table 1). Regarding the above results, we assume that some of the synthesized pyrazolopyrimidine derivatives could possibly have a promising effect against COVID-19, but this eventuality obviously requires serious in-depth and experimental scientific assays.

### 2.3. Prediction of Drug-Likeness Descriptors and Toxicity

#### 2.3.1. Predicted Molecular Properties and Drug-Likeness

The potential inhibitors or the docked ligands (tested molecules) can be selected based on their binding energies. Nevertheless, the ADMET (Absorption, Distribution, Metabolism, Excretion, and Toxicity) properties are considered as key steps and the appropriate methodologies employed in drug development and discovery processes [41]. In addition, the physicochemical properties are important parameters of a molecule that influence efficacy, safety, or metabolism and can be predicted by using Lipinski’s rule of five (RO5),as summarized in Table 2 and Table 3. The rule of five (RO5) deals with the dependency of active compounds and defines the twelve simple pharmacokinetics parameters named as the molecular weight, log P, H-bond donors, H-bond acceptors, octanol/water partition coefficient, number of rotatable bonds, topological polar surface area, molecular volume, percentage of absorption, N violation Lipinski, Lipinski Rule, and Pfizer Rule. These pharmacokinetic parameters are associated with the acceptable aqueous solubility and the intestinal permeability and comprise the first steps in oral bioavailability. The RO5 helps in predicting the medicinal and combinatorial chemistry of the selected class of molecules. The predicted values of various properties categorized as molecular weight describe the size of the molecule. Further, the value of log P corresponds to the lipophilicity of the molecules which is related to the solubility of the drug molecule in the aqueous medium. Therefore, the higher the solubility, the higher the activity of the therapeutic agents. Furthermore, the parameters of the H-bond donor and H-bond acceptor indicate the quantification of all atoms (N, O) and their efficiency in the formation of the hydrogen bond. The results obtained from the ADMET analysis with ADMETlab 2.0 software revealed that the predicted compounds **5** to **11** fully obeyed Lipinski’s rule of five while the compounds **12** and **13**, with an acid function, gave lightly elevated values of nHA, TPSA, and relatively low %Abs (Table 2). These findings suggest that the synthesized compounds are feasible in order to serve as a future potential antiviral candidate in the treatment of COVID-19 infections.

#### 2.3.2. Toxicity Analysis

**Figure 7:**
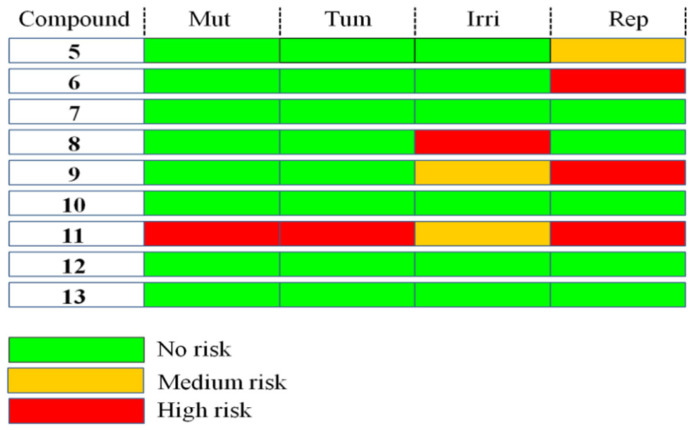
Computational Toxicity Risk plots of compounds **5**–**13**. Mut: Mutagenicity, Tum: Tumorigenicity, Irri: Irritation, Rep: Reproductive or developmental toxicity.

The toxicity determination of the selected class of compounds is another significant property in drug designing. Indeed, the computational approach to evaluate toxicity helps to determine the toxic level of doses. Therefore, it helps in the reduction of the risk of failure in the experimental procedure [42]. As depicted in Figure 7, it was found that almost all of the predicted compounds presented no risk of toxicity (mutagenicity, tumorigenicity, irritation, and reproductive effect), thereby suggesting the strong applicability of these compounds as antivirals to treat SARS-CoV-2, with the exception of the ligand **11** due, perhaps, to the presence of two chlorine substituents in its structure.

## 3. Materials and Methods

### 3.1. Chemistry

#### 3.1.1. General Procedure for the Synthesis of 3,6-dimethyl-1-phenyl-1,5-dihydro-4*H*-pyrazolo [3,4-*d*]pyrimidin-4-one (**2**)

In a 50 mL three-necked flask, 0.39 g (2 mmol) of aminopyrazole **1** is introduced in 10 mL of acetic anhydride. Then, 5 mL of phosphoric acid is added dropwise. The mixture is then brought to reflux for 3 h with magnetic stirring. The progress of the reaction is monitored by TLC (eluent 2: 8 ethyl acetate/chloroform), which shows the appearance of a new stain more polar than that of aminopyrazole1. Once cooled, ice water is added to the reaction crude, the precipitate is recovered by filtration, then it is washed with cold ether, dried, and then recrystallized from ethanol in order to give pyrimidinone **2**.

Yield: 79%; R_f_ = 0.52 (SiO_2_, CHCl_3_/MeOH, 95:5); m.p. 316–318 °C; UV-Vis (MeOH): λ_max_(log ε) = 234 nm (4.46), 272 nm (3.99); IR (ATR): $\tilde{\nu }$ = 2850m, 1668s, 1612m, 1591s, 1545w, 1531m, 1504s, 1435m, 1379m, 1321m, 1257m, 1181w, 1118m, 1079w, 1024w, 917m, 869m, 788m, 754s, 732s, 701w, 685s, 651s, 642s, 619m, 577s, 512m, 489m, 448m, 418w cm^−1^; ^1^H-NMR (500 MHz, DMSO-d6): δ = 12.22 (s, 1H, NH), 8.04 (dd, *J* = 7.7, 1.0 Hz, 2H, 2-H, 6-H), 7.53 (ddd, *J* = 8.6, 7.4, 1.1 Hz, 2H, 3-H, 5-H), 7.35 (tt, *J* = 7.2, 1.0 Hz, 1H, 4-H), 2.51 (d, *J* = 1.2 Hz, 3H, 13-H), 2.40 (d, *J* = 1.1 Hz, 3H, 12-H) ppm; ^13^C-NMR (126 MHz, DMSO-*d6*): δ = 158.6 (C-9), 158.6 (C-8), 152.9 (C-11), 145.7 (C-7), 138.4 (C-1), 129.0 (C-3, C-5), 126.4 (C-4), 121.3 (C-2, C-6), 103.7 (C-10), 21.5 (C-12), 13.3 (C-13) ppm; MS (ESI, negative mode, MeOH/DMSO (4:1)): *m*/*z* = 239 (100%, [M − H]^−^); analysis calcd for C_13_H_12_N_4_O (240.10): C 64.99, H 5.03, N 23.32; found: C 64.71, H 23.56, N 23.08.

#### 3.1.2. General Procedure for the Synthesis of Ethyl 2-(3,6-dimethyl-4-oxo-1-phenyl-1,4-dihydro-5*H*-pyrazolo[3,4-*d*]pyrimidin-5-yl)acetate (**3**)

The equimolar solution of 0.24 g (1 mmol) of pyrazolopyrimidinone **2**, 0.14 g (1 mmol) of anhydrous potassium carbonate and 0.12 g (1 mmol) of ethyl chloroacetate was refluxed in dry dimethylformamide (DMF) (60 mL) and stirred for 6 h, in a 100 mL three-necked flask. Once the starting material had disappeared, the reaction mixture was then cooled and poured into cold water and the formed precipitate was filtered off, then washed with water, dried, and finally recrystallized from ethanol in order to give the ester **3**.

Yield: 75%; R_f_ = 0.91 (SiO_2_, CHCl_3_/MeOH, 95:5); m.p. 122–124 °C; UV-Vis (MeOH): λ_max_(log ε) = 239 nm (4.49), 283 nm (3.79); IR (ATR): $\tilde{\nu }\;$ = 1762m, 1615m, 1509m, 1406m, 1377m, 1327m, 1296w, 1212s, 1151s, 1118m, 1053m, 791m, 758s, 716m, 683m, 512m cm^−1^; ^1^H-NMR (500 MHz, DMSO-*d6*): δ = 8.15 (d, *J* = 7.9 Hz, 2H, 6-H, 2-H), 7.55 (t, *J* = 7.8 Hz, 2H, 3-H, 5-H), 7.35 (t, *J* = 7.3 Hz, 1H, 4-H), 5.15 (s, 2H, 14-H), 4.20 (q, *J* = 7.1 Hz, 2H, 16-H), 2.61 (d, *J* = 2.9 Hz, 3H, 13-H), 2.58 (d, *J* = 2.6 Hz, 3H, 12-H), 1.22 (t, *J* = 7.1 Hz, 3H, 17-H) ppm; ^13^C-NMR (126 MHz, DMSO-*d6*): δ = 167.7 (C-15), 165.4 (C-9), 162.5 (C-11), 155.8 (C-8), 142.3 (C-7), 138.5 (C-1), 129.1 (C-3, C-5), 126.1 (C-4), 120.6 (C-2, C-6), 100.5 (C-10), 62.8 (C-14), 60.7 (C-16), 25.9 (C-12), 14.0 (C-17), 13.8 (C-13) ppm; MS (ASAP, positive mode, MeOH/DMSO (4:1)): *m*/*z* = 327 (100%, [M + H]^+^); analysis calcd for C_17_H_18_N_4_O_3_ (326.14): C 62.57, H 5.56, N 17.17; found: C 62.49, H 5.74, N 16.98.

#### 3.1.3. General Procedure for the Synthesis of 2-(3,6-dimethyl-4-oxo-1-phenyl-1,4-dihydro-5*H*-pyrazolo[3,4-*d*]pyrimidin-5-yl)acetohydrazide (**4**)

The previously synthesized ester-pyrazolopyrimidinone **3** (0.2 g, 0.6 mmol) was treated with an excess of hydrazine hydrate in a minimum amount of ethanol (5 mL) at room temperature for 1–2 h until a white precipitate formed. Then, the solid obtained was filtered off, washed with ethanol, and dried in order to obtain hydrazide **4**.

Yield: 65%; R_f_ = 0.14 (SiO_2_, CHCl_3_/MeOH, 95:5); m.p. 258–260 °C; UV-Vis (MeOH): λ_max_(log ε) = 234 nm (4.50), 279 nm (4.09); IR (ATR): $\tilde{\nu }$ = 3283m, 1706s, 1661s, 1566s, 1552s, 1497m, 1434w, 1383w, 1325w, 1277w, 1190w, 1127w, 994m, 950w, 869w, 786m, 758s, 725m, 705m, 687s, 654m, 618w, 570m, 551w, 513w, 498w cm^−1^; ^1^H-NMR (500 MHz, DMSO-*d6*): δ = 9.43 (s, 1H, NH), 8.05 (d, *J* = 8.1 Hz, 2H, 2-H, 6-H), 7.53 (t, *J* = 7.8 Hz, 2H, 3-H, 5-H), 7.35 (t, *J* = 7.4 Hz, 1H, 4-H), 4.72 (s, 2H, 14-H), 4.45–4.18 (m, 2H, NH2), 2.53 (s, 3H, 13-H), 2.50 (s, 3H, 12-H) ppm; ^13^C-NMR (126 MHz, DMSO-*d6*): δ = 166.1 (C-15), 159.8 (C-8), 157.7 (C-9), 150.8 (C-11), 145.9 (C-7), 138.3 (C-1), 129.1 (C-3, C-5), 126.5 (C-4), 121.1 (C-2, C-6), 102.9 (C-10), 44.2 (C-14), 23.6 (C-12), 13.2 (C-13) ppm; MS (ASAP, positive mode, MeOH/DMSO (4:1)): *m*/*z* = 313 (100%, [M + H]^+^); analysis calcd for C_15_H_16_N_6_O_2_ (312.12): C 57.68, H 5.16, N 26.91; found: C 57.55, H 5.39, N 26.77.

#### 3.1.4. General Procedure for the Synthesis of the Compounds **5** and **6**

A mixture of the appropriate hydrazide-pyrazolopyrimidinone **4** (0.31 g, 1 mmol) and 2,4-pentanedione (0.10 g, 1 mmol) or 1-phenyl-1,3-butanedione (0.16 g, 1 mmol) was stirred under reflux of dry dioxane (10 mL) in the presence of an a catalytic amount of acetic acid (0.001 mmol, 0.06 mL). Following a period of 8 h, the reaction mixture was cooled to room temperature, the dioxane was removed in vacuo, and the residue was filtered off, washed with ethanol, and dried in order to yield the compounds **5** and **6**, respectively.

*(5RS)-5-(2-(5-hydroxy-3,5-dimethyl-4,5-dihydro-1H-pyrazol-1-yl)-2-oxoethyl)-3,6-dimethyl-1-phenyl-1,5-dihydro-4H-pyrazolo[3,4-d]pyrimidin-4-one* (**5**). Yield: 75%; R_f_ = 0.52 (SiO_2_, CHCl_3_/MeOH, 95:5); m.p. 249–251 °C; UV-Vis (MeOH): λ_max_(log ε) = 235 nm (4.67), 278 nm (4.14); IR (ATR): $\tilde{\nu }$ = 3428w, 1700m, 1665s, 1595w, 1569m, 1555m, 1514w, 1435m, 1406w, 1379m, 1295m, 1197w, 1125w, 1094w, 1017w, 967w, 930w, 869m, 801w, 770m, 753w, 726w, 692m, 654m, 620w, 489w, 416w cm^−1^; ^1^H-NMR (500 MHz, DMSO-*d6*): δ = 8.07 (td, *J* = 7.7, 1.8 Hz, 2H, 2-H, 6-H), 7.55 (t, *J* = 8.0 Hz, 2H, 3-H, 5-H), 7.37 (tt, *J* = 7.7, 1.2 Hz, 1H, 4-H), 5.22–5.04 (m, 2H, 14-H), 3.00 (d, *J* = 18.6 Hz, 1H, 17-Ha), 2.85 (d, *J* = 18.7 Hz, 1H, 17-Hb), 2.53 (s, 3H, 13-H), 2.51 (s, 3H, 12-H), 2.05 (s, 3H, 19-H), 1.77 (s, 3H, 20-H) ppm; ^13^C-NMR (126 MHz, DMSO-*d6*): δ = 163.6 (C-15), 159.9 (C-16), 157.7 (C-9), 156.3 (C-8), 150.9 (C-11), 145.9 (C-7), 138.3 (C-1), 129.2 (C-3, C-5), 126.5 (C-4), 121.2 (C-2, C-6), 102.8 (C-10), 90.7 (C-18), 52.0 (C-17), 45.6 (C-14), 25.7 (C-20), 23.4 (C-12), 15.9 (C-19), 13.2 (C-13) ppm; MS (ESI, negative mode, MeOH/DMSO (4:1)): *m*/*z* = 393 (100%, [M − H]^−^); analysis calcd for C_20_H_22_N_6_O_3_ (394.18): C 60.90, H 5.62, N 21.31; found: C 60.43, H 5.81, N 21.19.

(*5RS)-5-(2-(5-hydroxy-3-methyl-5-phenyl-4,5-dihydro-1H-pyrazol-1-yl)-2-1-phenyl-1,5-dihydro-4H-pyrazolo[3,4-d]pyrimidin-4-one* (**6**). Yield: 82%; R_f_ = 0.65 (SiO_2_, CHCl_3_/MeOH, 95:5); m.p. 126–129 °C; UV-Vis (MeOH): λ_max_(log ε) = 235 nm (4.55), 279 nm (4.01); IR (ATR): $\tilde{\nu }$ = 1692m, 1672s, 1556m, 1511w, 1446m, 1385w, 1301m, 1209w, 1075w, 960w, 885w, 807w, 763m, 727w, 703m, 654w, 546w, 527w cm^−1^; ^1^H-NMR (500 MHz, DMSO-*d6*): δ = 8.02 (d, *J* = 7.8 Hz, 2H, 6-H), 7.53 (td, *J* = 8.5, 7.3 Hz, 2H, 5-H), 7.40–7.30 (m, 5H, 4-H, 21-H, 22-H, 24-H, 25-H), 7.25 (tt, *J* = 6.6, 1.4 Hz, 1H, 23-H), 7.03 (s, 1H, OH), 5.24 (s, 2H, 14-H), 3.24–3.09 (m, 2H, 17-H), 2.51 (s, 3H, 13-H), 2.45 (s, 3H, 12-H), 2.12 (s, 3H, 19-H) ppm; ^13^C-NMR (126 MHz, DMSO-*d6*): δ = 163.0 (C-15), 159.8 (C-16), 157.7 (C-9), 155.9 (C-11), 150.8 (C-8), 145.9 (C-20), 143.3 (C-7), 138.2 (C-1), 129.1 (C-3, C-5), 127.9 (C-22, C-24), 127.1 (C-4), 126.5 (C-23), 124.5 (C-21, C-25), 121.2 (C-2, C-6), 102.7 (C-10), 92.3 (C-18), 55.3 (C-17), 45.4 (C-14), 23.4 (C-12), 15.8 (C-19), 13.2 (C-13) ppm; MS (ESI, negative mode, MeOH/DMSO (4:1)): *m*/*z* = 455 (100%, [M − H]^−^); analysis calcd for C_25_H_24_N_6_O_3_ (456.19): C 65.78, H 5.30, N 18.41; found: C 65.47, H 5.51, N 18.23.

#### 3.1.5. General Procedure for the Synthesis of the Compounds **7**–**13**

A mixture of the appropriate hydrazide-pyrazolopyrimidinone **4** (0.31 g, 1 mmol) and cyclic anhydride (1 eq) was stirred under reflux of dry dioxane (10 mL) in the presence of a catalytic amount of acetic acid (0.001 mmol, 0.06 mL). Following a period of 8 h, the reaction mixture was cooled to room temperature, the dioxane was removed in vacuo and the residue was washed with ethanol, and dried in order to yield the compounds **7**–**13**, respectively.

*(3,6-dimethyl-4-oxo-1-phenyl-1,4-dihydro-5H-pyrazolo[3,4-d]pyrimidin-5-yl)-N-(1,3-dioxoisoindolin-2-yl)acetamide* (**7**). Yield: 87%; R_f_ = 0.34 (SiO_2_, CHCl_3_/MeOH, 95:5); m.p. 310–315 °C; UV-Vis (MeOH): λ_max_(log ε) = 218 nm (4.80), 280 nm (4.17); IR (ATR): $\tilde{\nu }\;$ = 3334w, 1795w, 1734s, 1714s, 1693s, 1566m, 1554m, 1510m, 1463w, 1439w, 1407w, 1368w, 1324w, 1219m, 1122m, 1086w, 1028w, 954w, 882m, 869w, 790w, 762m, 726m, 713m, 692m, 653w, 617w, 576w, 526w, 479s cm^−1^; ^1^H-NMR (500 MHz, DMSO-*d6*): δ = 11.26 (s, 1H, NH), 8.03 (d, *J* = 8.0 Hz, 2H, 2-H, 6-H), 7.98–7.87 (m, 4H, 18-H, 19-H), 7.52 (t, *J* = 7.8 Hz, 2H, 3-H, 5-H), 7.35 (t, *J* = 7.4 Hz, 1H, 4-H), 5.10 (s, 2H, 14-H), 2.61 (s, 3H, 13-H), 2.53 (s, 3H, 12-H) ppm; ^13^C-NMR (126 MHz, DMSO-*d6*): δ = 166.8 (C-15), 164.8 (C-16), 159.5 (C-9), 157.6 (C-8), 150.7 (C-11), 146.0 (C-7), 138.2 (C-1), 135.3 (C-19), 129.4 (C-17), 129.1 (C-3, C-5), 126.6 (C-4), 123.8 (C-18), 121.2 (C-6), 102.8 (C-10), 43.6 (C-14), 23.3 (C-12), 13.2 (C-13) ppm; MS (ESI, negative mode, MeOH/DMSO (4:1)): *m*/*z* = 441 (100%, [M − H]^−^); analysis calcd for C_23_H_18_N_6_O_4_ (442.14): C 62.44, H 4.10, N 19.00; found: C 62.15, H 4.37, N 18.76.

*2-(3,6-dimethyl-4-oxo-1-phenyl-1,4-dihydro-5H-pyrazolo[3,4-d]pyrimidin-5-yl)-N-(1,3-dioxo-1,3,4,5,6,7-hexahydro-2H-isoindol-2-yl)acetamide* (**8**). Yield: 45%; R_f_ = 0.38 (SiO_2_, CHCl_3_/MeOH, 95:5); m.p. 266 °C; UV-Vis (MeOH): λ_max_(log ε) = 230 nm (4.55), 279 nm (4.03); IR (ATR): $\tilde{\nu }$ = 3316w, 2943w, 1732s, 1709s, 1679s, 1596w, 1557s, 1512m, 1429m, 1361w, 1327w, 1270w, 1228m, 1192m, 1122w, 1070w, 966w, 870w, 796m, 774m, 714m, 704m, 697m, 620w, 543m, 467m cm^−1^; ^1^H-NMR (500 MHz, DMSO-d6): δ = 10.93 (s, 1H, NH), 8.04 (d, *J* = 7.8 Hz, 2H, 2-H, 6-H), 7.54 (t, *J* = 7.8 Hz, 2H, 3-H, 5-H), 7.37 (t, *J* = 7.4 Hz, 1H, 4-H), 5.03 (s, 2H, 14-H), 2.57 (s, 3H, 13-H), 2.54 (s, 3H, 12-H), 2.33–2.23 (m, 4H, 18-H), 1.74–1.67 (m, 4H, 19-H) ppm; ^13^C-NMR (126 MHz, DMSO-d6): δ = 167.7 (C-8), 166.7 (C-15), 159.5 (C-16), 157.6 (C-9), 150.7 (C-11), 146.0 (C-7), 140.5 (C-17), 138.2 (C-1), 129.1 (C-3, C-5), 126.6 (C-4), 121.2 (C-2, C-6), 102.8 (C-10), 43.5 (C-14), 23.2 (C-12), 20.6 (C-19), 19.6 (C-18), 13.2 (C-13) ppm; MS (ESI, negative mode, MeOH/DMSO (4:1)): *m*/*z* = 445 (100%, [M − H]^−^); analysis calcd for C_23_H_22_N_6_O_4_ (446.17): C 61.88, H 4.97, N 18.82; found: C 61.60, H 5.18, N 18.64.

*2-(3,6-dimethyl-4-oxo-1-phenyl-1,4-dihydro-5H-pyrazolo[3,4-d]pyrimidin-5-yl)-N-((3aR,4R,7S,7aS)-1,3-dioxo-1,3,3a,4,7,7a-hexahydro-2H-4,7-methanoisoindol-2-yl)acetamide* (**9**). Yield: 82%; R_f_ = 0.25 (SiO_2_, CHCl_3_/MeOH, 95:5); m.p. 235–238 °C; UV-Vis (MeOH): λ_max_(log ε) = 234 nm (4.34), 279 nm (3.96); IR (ATR): $\tilde{\nu }$ = 3195w, 3023w, 1783w, 1723s, 1664s, 1594w, 1554m, 1510m, 1441w, 1419w, 1376w, 1324w, 1214m, 1194s, 1120w, 1083w, 1033w, 972w, 870m, 843w, 796w, 751m, 730m, 688m, 653w, 612w, 540w, 464w cm^−1^; ^1^H-NMR (500 MHz, DMSO-*d6*): δ = 8.05 (d, *J* = 7.4 Hz, 2H, 2-H, 6-H), 7.55 (t, *J* = 8.0 Hz, 2H, 3-H, 5-H), 7.37 (tt, *J* = 7.0, 1.1 Hz, 1H, 4-H), 6.18–5.95 (m, 2H, 19-H), 3.58 (s, 2H, 14-H), 3.47 (m, 2H, 18-H), 3.30 (m, 2H, 17-H), 2.55 (s, 3H, 12-H), 2.53 (s, 3H, 13-H), 1.56 (t, *J* = 10.6 Hz, 2H, 20-H) ppm; ^13^C-NMR (126 MHz, DMSO-*d6*): δ = 174.2 (C-8), 165.6 (C-15), 160.0 (C-16), 158.0 (C-9), 151.2 (C-11), 146.5 (C-7), 138.6 (C-1), 134.9 (C-19), 129.6 (C-5), 127.1 (C-4), 121.7 (C-6), 103.3 (C-10), 66.8 (C-14), 51.7 (C-20), 44.8 (C-17), 44.0 (C-18), 23.7 (C-12), 13.7 (C-13) ppm; MS (ESI, negative mode, MeOH/DMSO (4:1)): *m*/*z* = 457 (100%, [M − H]^−^); analysis calcd for C_24_H_22_N_6_O_4_ (458.17): C 62.87, H 4.84, N 18.33; found: 62.73, H 5.03, N 18.04.

*2-(3,4-dimethyl-2,5-dioxo-2,5-dihydro-1H-pyrrol-1-yl)-2-(3,6-dimethyl-4-oxo-1-phenyl-1,4-dihydro-5H-pyrazolo[3,4-d]pyrimidin-5-yl)acetamide* (**10**). Yield: 57%; R_f_ = 0.36 (SiO_2_, CHCl_3_/MeOH, 95:5); m.p. 303–306 °C; UV-Vis (MeOH): λ_max_(log ε) = 229 nm (4.62), 278 nm (4.10); IR (ATR): $\tilde{\nu }$ = 3242w, 3038w, 1731s, 1696s, 1589w, 1565m, 1555m, 1539m, 1494m, 1436m, 1387m, 1321w, 1243m, 1232m, 1191m, 1089m, 1071w, 982w, 952m, 869m, 793m, 759s, 721s, 691s, 661m, 652m, 542w, 525m, 516m, 508m, 405m cm^−1^; ^1^H-NMR (500 MHz, DMSO-*d6*): δ = 10.93 (s, 1H, NH), 8.02 (dt, *J* = 7.6, 1.6 Hz, 2H, 2-H, 6-H), 7.51 (t, *J* = 8.0 Hz, 2H, 3-H, 5-H), 7.34 (t, *J* = 7.5 Hz, 1H, 4-H), 5.00 (s, 2H, 14-H), 2.54 (s, 3H, 13-H), 2.51 (s, 3H, 12-H), 1.93 (s, 6H, 18-H) ppm; ^13^C-NMR (126 MHz, DMSO-*d6*): δ = 168.8 (C-8), 166.7 (C-15), 159.5 (C-16), 157.6 (C-9), 150.7 (C-11), 146.0 (C-7), 138.2 (C-1), 136.6 (C-17), 129.1 (C-5), 126.6 (C-4), 121.2 (C-6), 102.8 (C-10), 43.5 (C-14), 23.2 (C-12), 13.2 (C-13), 8.6 (C-18) ppm; MS (ESI, negative mode, MeOH/DMSO (4:1)): *m*/*z* = 419 (100%, [M − H]^−^); analysis calcd for C_21_H_20_N_6_O_4_ (420.15): C 59.99, H 4.80, N 19.99; found: C 59.75, H 5.02, N 19.85.

*(3,4-dichloro-2,5-dioxo-2,5-dihydro-1H-pyrrol-1-yl)-2-(3,6-dimethyl-4-oxo-1-phenyl-1,4-dihydro-5H-pyrazolo[3,4-d]pyrimidin-5-yl)acetamide* (**11**). Yield: 66%; R_f_ = 0.05 (SiO_2_, CHCl_3_/MeOH, 9:1); m.p. > 260 °C (slow decomp.); UV-Vis (MeOH): λ_max_(log ε) = 234 nm (4.50), 380 nm (4.08); IR (ATR): $\tilde{\nu }$ = 3239br, 1677m, 1561s, 1510m, 1374m, 1325w, 1234w, 1192w, 1093w, 1029w, 870w, 791m, 757m, 689m, 653m, 508w cm^−1^; ^1^H-NMR (300 MHz, DMSO-*d6*): δ = 10.65 (s, 1H, NH), 8.05 (d, *J* = 7.77Hz,2H, 2-H, 6-H), 7.54 (t, *J* = 7.47 Hz, 2H, 3-H, 5-H), 7.36 (t, *J* = 7.02 Hz, 1H, 4-H), 4.90 (s, 2H, 14-H), 2.56–2.52 (m, 6H, 12-H, 13-H) ppm; ^13^C-NMR (126 MHz, DMSO-*d6*): δ = 168.4 (C-8), 164.4 (C-15), 159.8 (C-16), 157.7 (C-9), 150.9 (C-11), 146.0 (C-7), 138.3 (C-1), 129.2 (C-3, C-5), 126.6 (C-4), 124.2 (C-17), 121.3 (C-2, C-6), 102.9 (C-10), 43.9 (C-14), 23.4 (C-12), 13.2 (C-13) ppm; MS (ESI, negative mode, MeOH/DMSO (4:1)): *m*/*z* = 433 (100%, [M − HCN]^−^); analysis calcd for C_19_H_14_N_6_O_4_C_l2_ (460.05): C 49.48, H 3.06, N 18.22; found: C 49.20, H 3.32, N 17.95.

*(E)-4-(2-(2-(3,6-dimethyl-4-oxo-1-phenyl-1,4-dihydro-5H-pyrazolo[3,4-d]pyrimidin-5-yl)acetyl)hydrazinyl)-4-oxobut-2-enoic acid* (**12**). Yield: 66%; R_f_ = 0.03 (SiO_2_, CHCl_3_/MeOH, 9:1); m.p. 265–270 °C; UV-Vis (MeOH): λ_max_(log ε) = 234 nm (4.43), 279 nm (4.09); IR (ATR): $\tilde{\nu }$ = 3240br, 1682m, 1557s, 1505m, 1433m, 1384w, 1325w, 1235w, 1192w, 1126w, 1030w, 960w, 910w, 869w, 790w, 756m, 689w, 653w, 618w cm^−1^; ^1^H-NMR (300 MHz, DMSO-*d6*): δ = 15.86 (s, 1H, OH),10.31 (s, 1H, NH), 9.33 (s, 1H, NH), 8.07–8.05 (m, 2H, 2-H, 6-H), 7.56–7.51 (m, 2H, 3-H, 5-H), 7.35 (t, *J* = 7.2 Hz, 1H, 4-H), 6.20–5.86 (m, 2H, 17-H, 18-H), 4.92–5.12 (m, 2H, 14-H), 2.55–2.52 (m, 6H, 12-H, 13-H) ppm; ^13^C-NMR (126 MHz, DMSO-*d6*): δ = 168.0 (C-15), 160.0 (C-16), 160.0 (C-19), 157.8 (C-8), 157.8 (C-9), 150.9 (C-11), 146.0 (C-7), 138.3 (C-1), 136.1 (C-17), 129.2 (C-3, C-5), 129.0 (C-18), 126.5 (C-4), 121.2 (C-6), 102.9 (C-10), 44.5 (C-14), 23.6 (C-12), 13.2 (C-13) ppm; MS (ESI, negative mode, MeOH/DMSO (4:1)): *m*/*z* = 409 (100%, [M − H]^−^); analysis calcd for C_19_H_18_N_6_O_5_ (410.13): C 55.61, H 4.42, N 20.48; found: C 55.44, H 4.69, N 20.16.

*4-(2-(2-(3,6-dimethyl-4-oxo-1-phenyl-1,4-dihydro-5H-pyrazolo[3,4-d]pyrimidin-5-yl)acetyl)hydrazinyl)-4-oxobutanoic acid* (**13**). Yield: 60%; R_f_ = 0.2 (SiO_2_, CHCl_3_/MeOH, 9:1); m.p. 234–237 °C; UV-Vis (MeOH): λ_max_(log ε) = 234 nm (4.44), 279 nm (4.01); IR (ATR): $\tilde{\nu }$ = 3208w, 1703s, 1611s, 1551m, 1496m, 1439m, 1406m, 1379w, 1350w, 1263w, 1188m, 1030w, 987w, 951m, 866w, 789m, 756m, 688w, 651m, 586w, 506w, 448m cm^−1^; ^1^H-NMR (500 MHz, DMSO-*d6*): δ = 15.85 (s, 1H, OH), 10.26 (s, 1H, NH), 9.91 (s, 1H, NH), 8.02 (d, *J* = 8.0 Hz, 2H, 2-H, 6-H), 7.51 (t, *J* = 7.8 Hz, 2H, 3-H, 5-H), 7.33 (t, *J* = 7.5 Hz, 1H, 4-H), 4.82 (s, 2H, 14-H), 2.53 (s, 3H, 13-H), 2.48 (s, 3H, 12-H), 2.45–2.33 (m, 4H, 17-H, 18-H) ppm; ^13^C-NMR (126 MHz, DMSO-*d6*): δ = 174.0 (C-19), 170.6 (C-16), 166.3 (C-15), 160.2 (C-8), 158.1 (C-9), 151.3 (C-11), 146.4 (C-7), 138.7 (C-1), 129.6 (C-3, C-5), 127.0 (C-4), 121.6 (C-2, C-6), 103.3 (C-10), 44.3 (C-14), 29.2 (C-18), 28.4 (C-17), 23.9 (C-12), 13.6 (C-13) ppm; MS (ESI, negative mode, MeOH/DMSO (4:1)): *m*/*z* = 411 (100%, [M − H]^−^); analysis calcd for C_19_H_20_N_6_O_5_ (412.15): C 55.34, H 4.89, N 20.38; found: C 55.06, H 5.14, N 20.03.

The NMR spectra of the compounds **2**–**13** are given as Supplementary Materials.

### 3.2. Molecular Docking Procedure

The molecular docking simulations were performed using the Auto Dock 4.2 program package [43]. The optimization of the geometries of the compounds was carried out using ACD (3D viewer) software (http://www.filefacts.com/acd3d-viewer-freeware-info (accessed on 10 July 2022)). The three dimensional structure of PDB (PDB: 7TLL) was obtained from the RSCB Protein Data Dank [44]. First, the water molecules were eliminated and the missing hydrogens and Gasteiger charges were then added to the system during the preparation of the receptor input file. Once completed, the AutoDock Tools were used for the preparation of the corresponding ligand and protein files (PDBQT). Next, the pre-calculation of the grid maps was performed using Auto Grid in order to save a lot of time during the docking. Subsequently, the docking calculation was carried out using a grid per map with 40 × 40 × 40 Å points of (PDB: 7TLL) in addition to a grid-point spacing of 0.375 Å, which was centered on the receptor in order to determine the active site. Finally, the visualization and analysis of the interactions were performed using Discovery Studio 2017R2 (https://www.3dsbiovia.com/products/collaborative-science/biovia-discovery-studio/ (accessed on 10 July 2022)).

### 3.3. Chemoinformatics

#### 3.3.1. ADMET Properties

In order to predict the pharmacokinetic properties such as: molecular weight, number of hydrogen-bond acceptors, number of hydrogen-bond donors, octanol/water partition coefficient, number of rotatable bonds, topological polar surface area, molecular volume, percentage of absorption, N violation Lipinski, Lipinski Rule, and Pfizer Rule of all synthesized compounds, the chemoinformatics tools (database): ADMETlab 2.0 (https://admetmesh.scbdd.com/service/evaluation/cal/ (accessed on 15 July 2022)) and Molinspiration (https://www.molinspiration.com/ (accessed on 15 July 2022)) were used.

#### 3.3.2. Toxicity Prediction

Toxicity issues remain the important aspect to be resolved, therefore, the toxicity profile in terms of the various parameters such as mutagenicity, tumorigenicity, irritation, and reproductive or developmental toxicity of the synthesized compounds were determined using the free online available software named the OSIRIS Property Explorer (https://www.organic-chemistry.org/prog/peo/(accessed on 15 July 2022)).

## 4. Conclusions

In summary, a new series of pyrazole, 2,5-pyrrolidinedione, and carboxylic acid linked pyrazolopyrimidinones were synthesized and evaluated theoretically in a silico study of molecular docking and ADMET properties in order to predict their inhibition of M^pro^ which characterizes the Omicron variant designed for SARS-CoV-2. Significant results were obtained, and some of these compounds, such as **7**, **8**, and **13**, showed interesting binding energies and types of interactions compared to nirmatrelvir that was employed as a reference drug. These results showed that pyrazolopyrimidinone linked to 2,5-pyrrolidinedione and the unconjugated carboxylic acid could have interesting applications against the Omicron variant of SARS-CoV-2, and therefore encourages to expand this series via the synthesis of more analogues, and to test them all experimentally in order to draw the right conclusions.

## Figures and Tables

**Table 1:** Ligand and receptor (Omicron) interactions: binding affinity, number of conventional hydrogen bonds and interacting amino acid residues.

Ligand	Binding Affinity (kcal/mol)	Intermolecular Interactions	
		Conventional Hydrogen Bonds	Interacting Amino Acid Residues
**5**	−7.5	1	HIS41, MET49, MET165, GLU166 *, LEU167, PRO168, ASP187, GLN189
**6**	−7.7	3	MET49, CYS145 *, HIS163, HIS164 *, GLU166 *
**7**	−8.0	2	HIS41, ASN142 *, GLY143, CYS145 *, MET165, PRO168, GLN189, THR190, ALA191
**8**	−8.2	3	THR26 *, HIS41, MET49, GLY143 *, CYS145, HIS163, MET165, GLU166 *
**9**	−7.4	2	HIS41, MET49, ASN142, CYS145, MET165, GLU166 **, PRO168
**10**	−7.7	3	HIS41, MET49, ASN142, CYS145, GLU166 **, THR190 *
**11**	−7.6	3	HIS41, MET49, GLY143, CYS145, GLU166 **, THR190 *
**12**	−7.0	1	HIS41, MET49, GLY143, CYS145, GLU166 *
**13**	−7.8	3	HIS41, MET49, ASN142, CYS145, GLU166 ***
**Nirmatrelvir** ^R^	−7.7	2	HIS41, MET49, PHE140, LEU141, SER144 *, HIS163, MET165, GLU166 *

^R^: Reference drug; *: One H-bond; **: Two H-bonds; ***: Three H-bonds.

**Table 2:** Predicted physicochemical properties for the target compounds **5**–**13**.

Compd. No.	MW ^a^	nHA ^b^	nHD ^c^	logP(*o*/*w*) ^d^	Nrotbo ^e^	TPSA ^f^	MV ^g^	%Abs ^h^
Acceptable Value	<500	<10	<5	<5	≤10	<140	500	100%
**5**	394.180	9	1	0.654	4	105.610	388.873	72.56
**6**	456.190	9	1	1.515	5	105.610	458.888	72.56
**7**	442.140	10	1	1.101	5	119.190	430.449	67.87
**8**	446.170	10	1	1.327	5	127.200	435.722	65.11
**9**	458.170	10	1	1.014	5	127.200	444.462	65.11
**10**	420.150	10	1	0.902	5	119.190	409.687	67.87
**11**	460.050	10	1	1.263	5	119.190	405.517	67.87
**12**	410.130	11	3	−0.505	8	148.870	392.441	57.63
**13**	412.150	11	3	−0.562	9	148.210	395.078	57.86

But this ^a^ Molecular weight, ^b^ Number of hydrogen-bond acceptors, ^c^ Number of hydrogen-bond donors, ^d^ Octanol/water partition coefficient, ^e^ Number of rotatable bonds, ^f^ Topological polar surface area, ^g^ Molecular volume, and ^h^ Percentage of absorption (%Abs = 109 − [0.345 × TPSA]).

**Table 3:** Predicted medicinal chemistry properties and the Lipinski parameters for the target compounds **5**–**13**.

Compd. No.	5	6	7	8	9	10	11	12	13
**Pfizer Rule**	+	+	+	+	+	+	+	+	+
**Lipinski Rule**	+	+	+	+	+	+	+	+	+
**^i^ Nviolation Lipinski** **(Acceptable Value ≤ 1)**	0	0	0	0	0	0	0	1	1

The pred +: Accepted; ^i^ Nviolation Lipinski: number of violations according to the Lipinski “rule of five”.

## Data Availability

The source data for the underlying tables and figures are available from the authors upon request.

## Supplementary Materials

The following are available online at https://www.mdpi.com/article/10.3390/molecules27165303/s1, depicted NMR (^1^H, ^13^C) spectra of the compounds **2**–**13**.

## Sample Availability

Samples of the compounds are available from the authors.
