# Supplementary material for: Synthesis and In Silico Docking Study towards M-Pro of Novel Heterocyclic Compounds Derived from Pyrazolopyrimidinone as Putative SARS-CoV-2 Inhibitors
Source: Molecules. 2022 Aug 19;27(16):5303. doi: 10.3390/molecules27165303 (PMC9416631; doi:10.3390/molecules27165303)

molecules

Supplementary Materials

Synthesis and In Silico Docking Study towards M-Pro of Novel Heterocyclic Compounds Derived from Pyrazolopyrimidinone as Putative SARS-CoV-2 Inhibitors

Mabrouk Horchani [1], Niels V. Heise [2], René Csuk [2,*], Hichem Ben Jannet [1,*], Abdel Halim Harrath [3] and Anis Romdhane [1]

[1] Laboratory of Heterocyclic Chemistry, Natural Products and Reactivity, Medicinal Chemistry and Natural Products (LR11ES39), Faculty of Sciences of Monastir, University of Monastir, Monastir 5000, Tunisia
[2] Department of Organic Chemistry, Martin-Luther-University Halle-Wittenberg, Kurt-Mothes-Str. 2, D-06120 Halle, Germany
[3] College of Science, Department of Zoology, King Saud University, Riyad 11451, Saudi Arabia
* Correspondence: rene.csuk@chemie.uni-halle.de (R.C.); hichem.bjannet@gmail.com (H.B.J.)

General procedures

TLC was carried out on silica gel (Macherey-Nagel, detection under UV light and with cerium molybdate reagent). The used solvents were dried according to usual procedures. Melting points are uncorrected (Leica hot stage microscope, or BUCHI melting point M-565). IR spectra were recorded on a Perkin Elmer FT-IR spectrometer Spectrum 1000 or on a Perkin-Elmer Spectrum Two (UATR Two Unit). NMR spectra were recorded using the Agilent spectrometers DD2 500 MHz and VNMRS 400 MHz (d given in ppm, J in Hz; typical experiments: APT, H-H-COSY, HMBC, HSQC, NOESY), MS spectra were taken on an Advion Expression CMS instrument, and elemental analyses were conducted on a Foss-Heraeus Vario EL (CHNS) unit.

5-amino-3-methyl-1-phenyl-1H-pyrazole-4-carbonitrile (1)

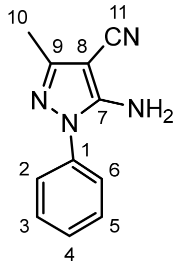

Has already been investigated with reference:

M. Horchani et al., (2021), Int J Mol Sci., 22(19):10258.

doi: 10.3390/ijms221910258.

Compound (2)

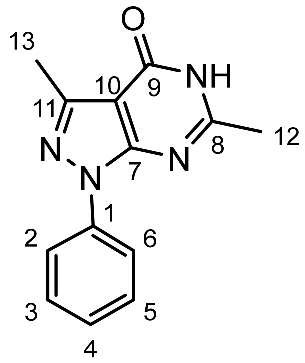

NMR Spectra of **2**

^{1}H-NMR (500 MHz, d-DMSO)

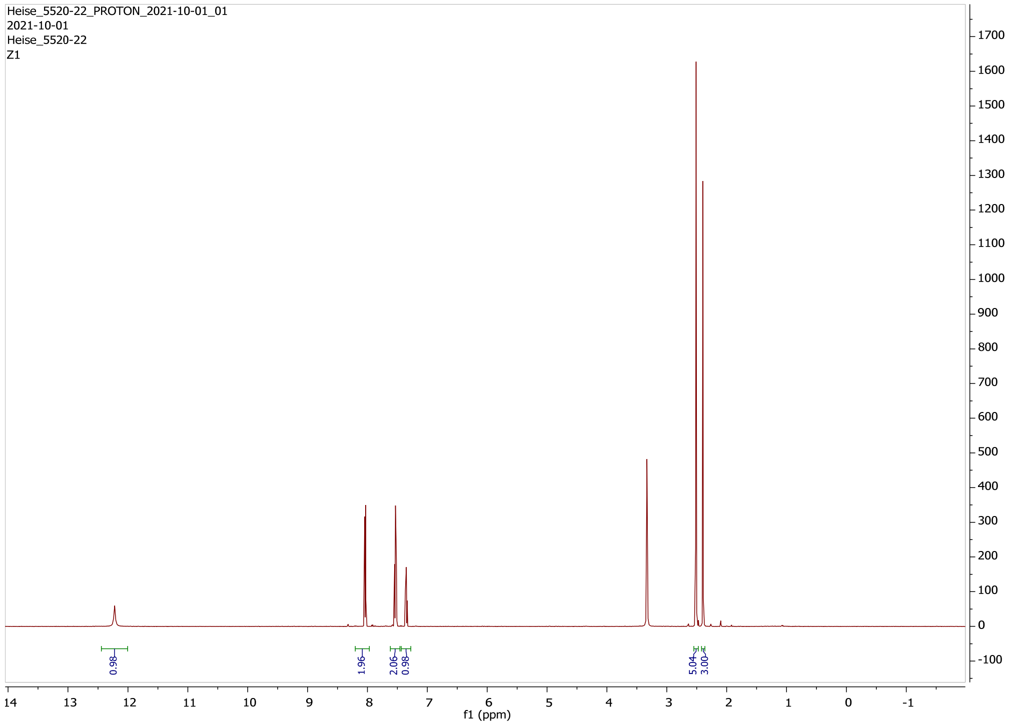

^{13}C-NMR (126 MHz, d-DMSO)

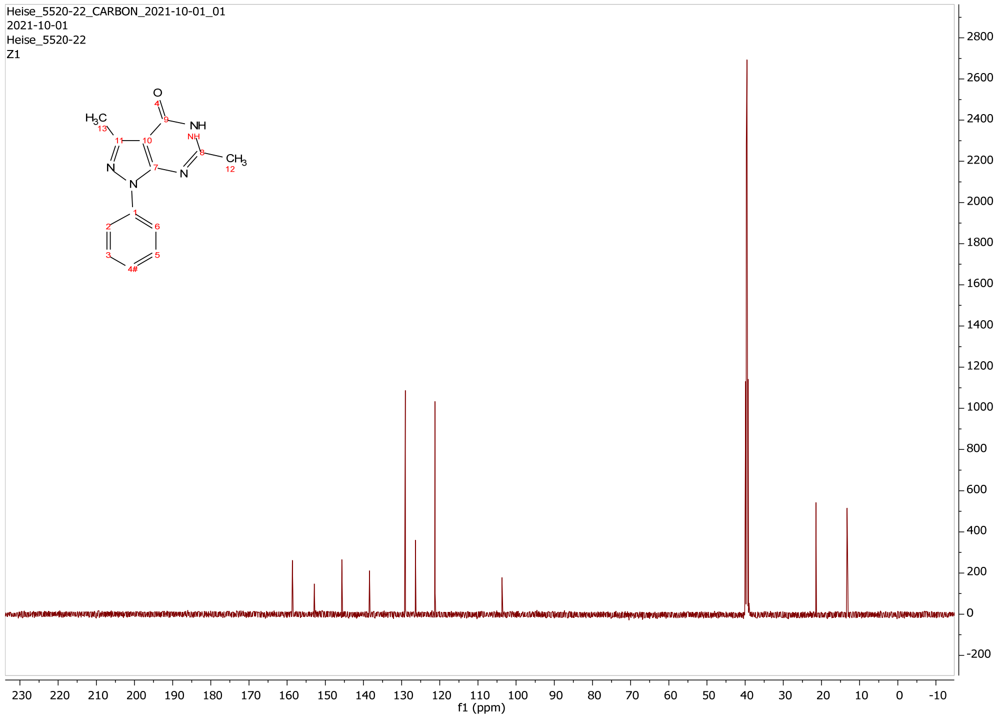

Compound (3)

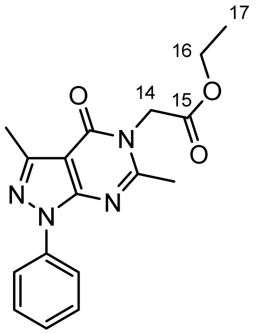

NMR Spectra of **3**

¹H-NMR (500 MHz, d-DMSO)

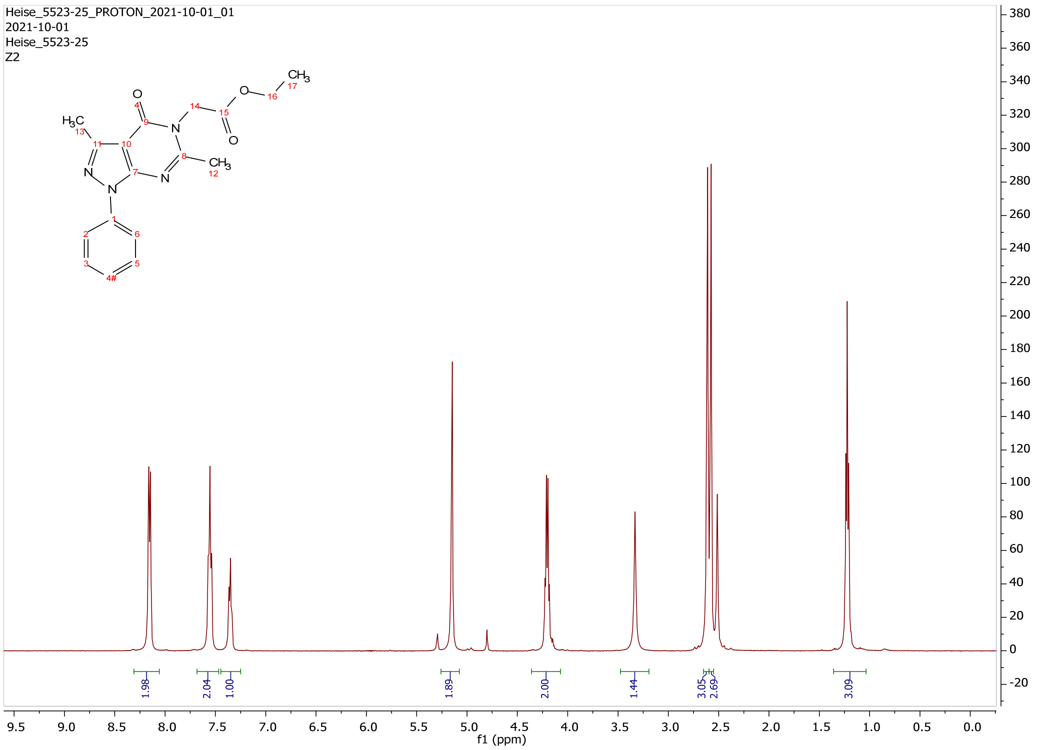

¹³C-NMR (126 MHz, d-DMSO)

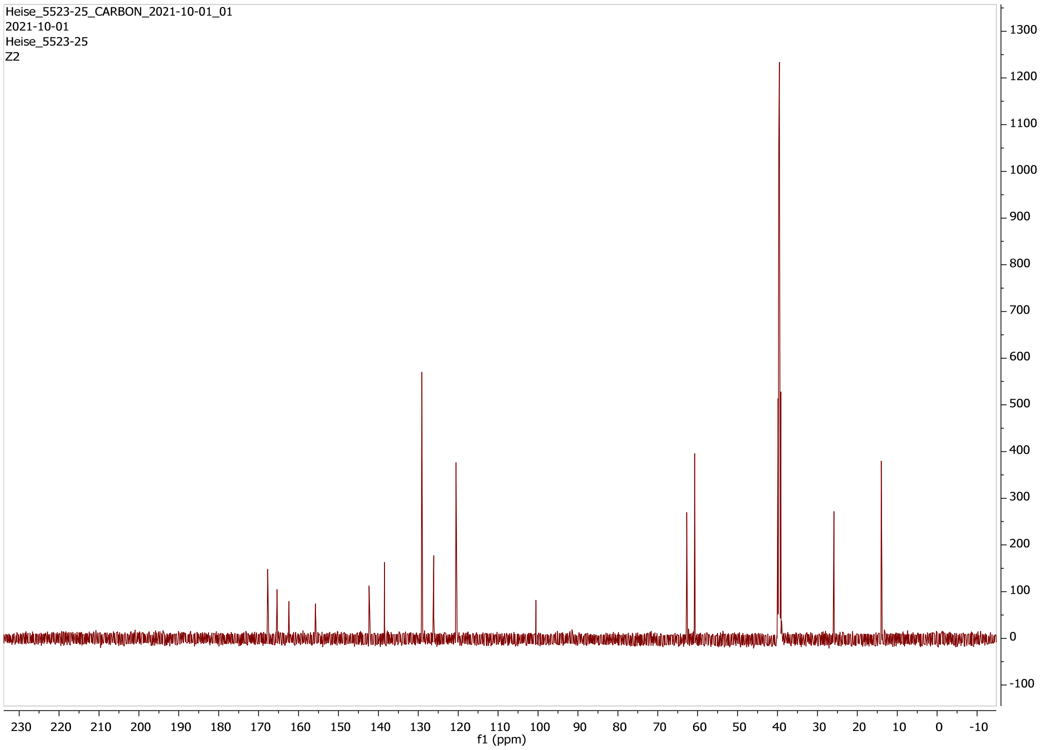

Compound **(4)**

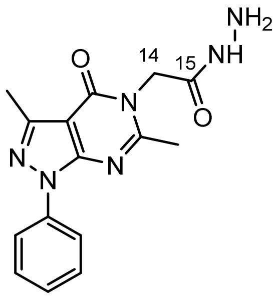

NMR Spectra of **4**

^{1}H-NMR (500 MHz, d-DMSO)

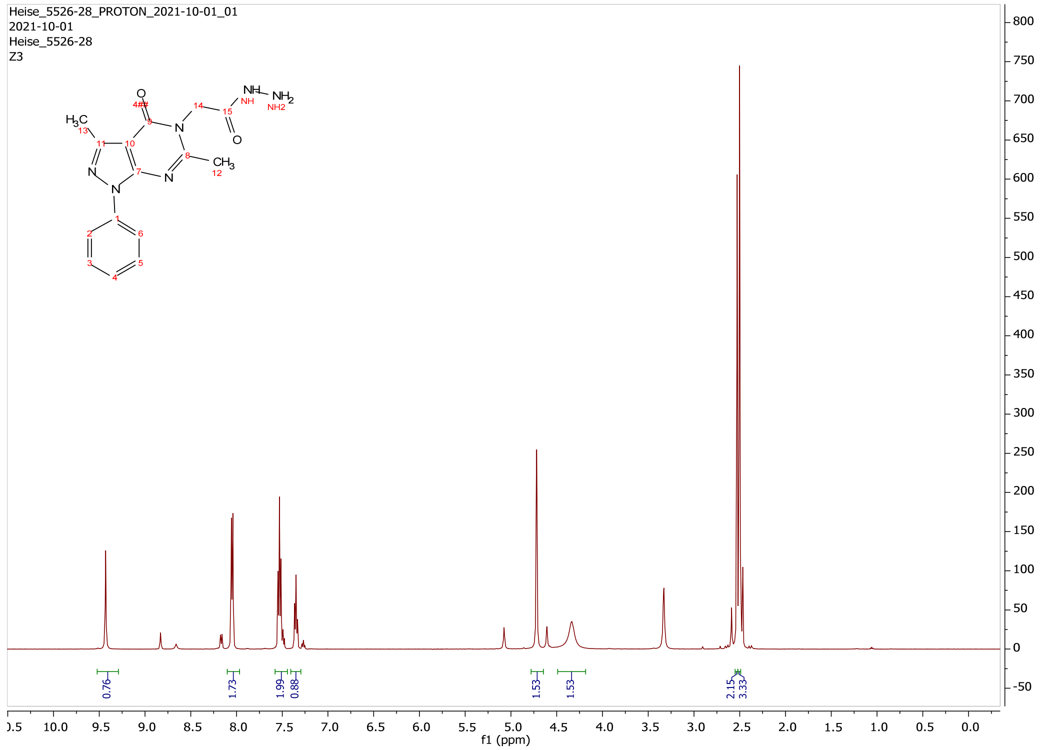

^{13}C-NMR (126 MHz, d-DMSO)

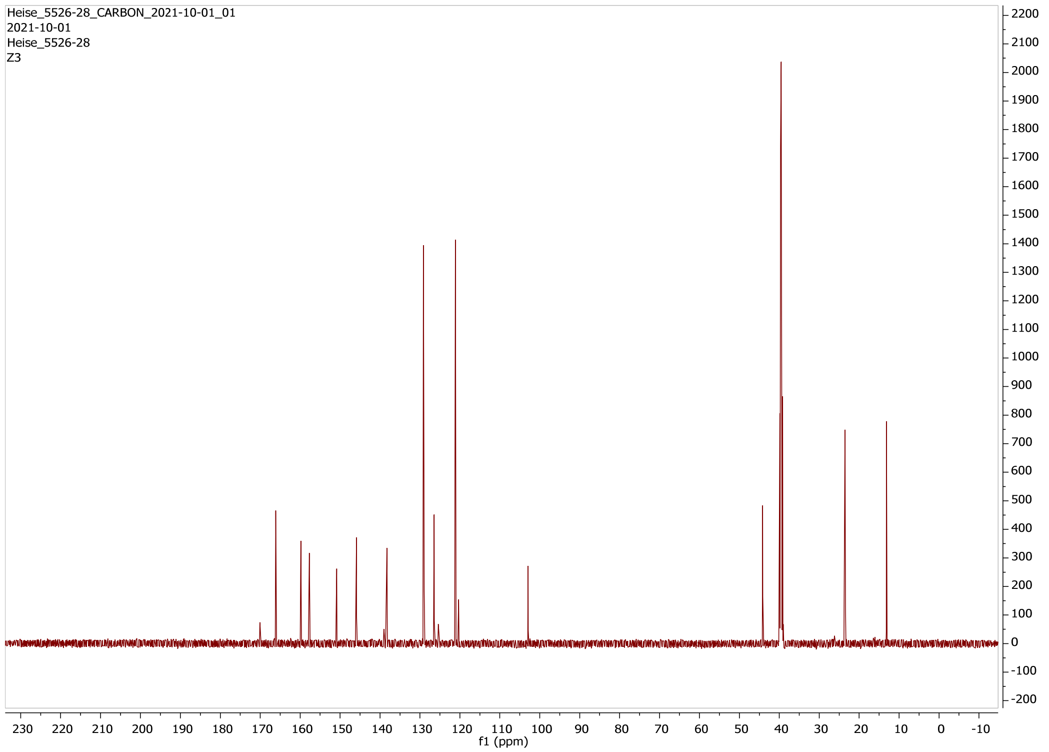

Compound **(5)**

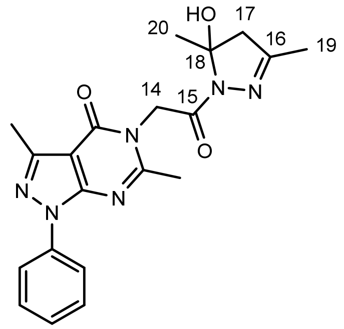

NMR Spectra of **5**

^{1}H-NMR (500 MHz, d-DMSO)

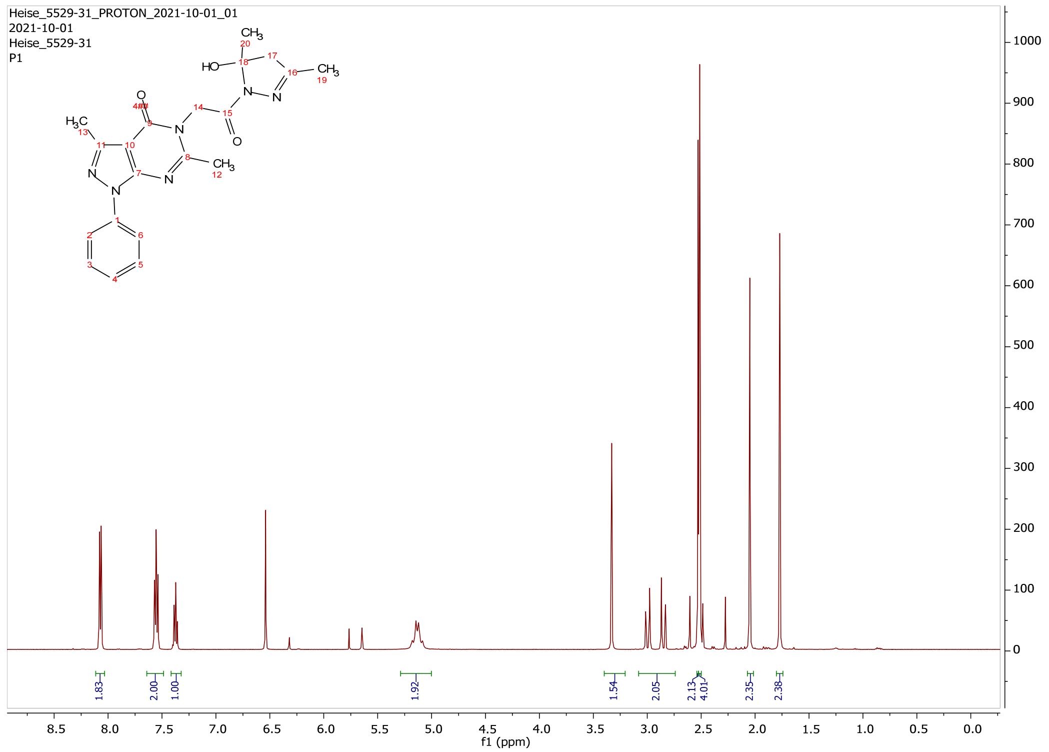

Expansions of the ^{1}H NMR

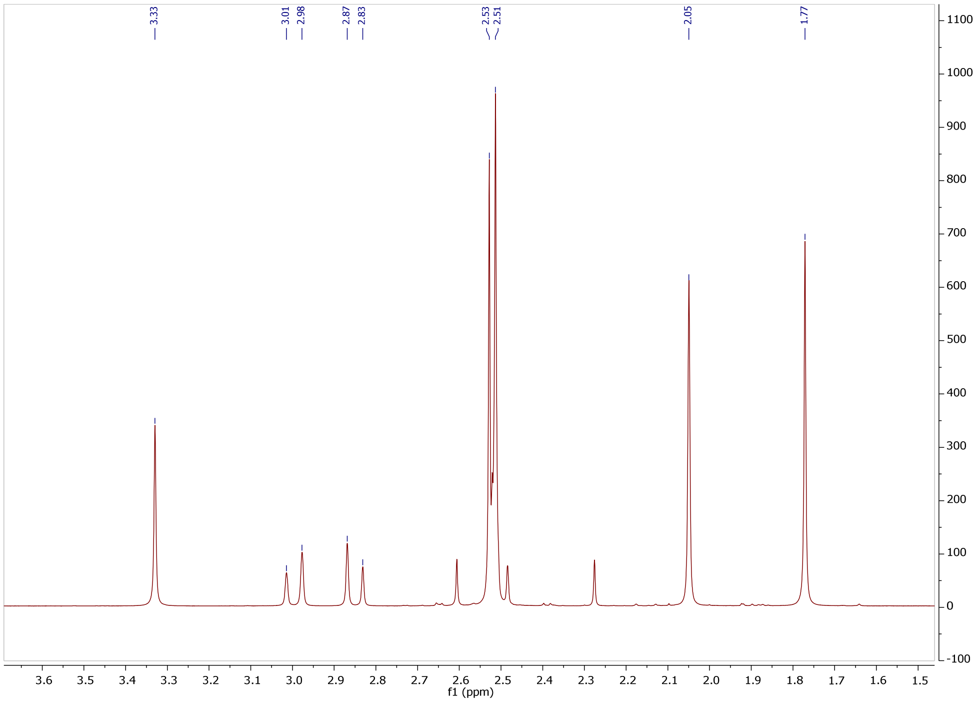

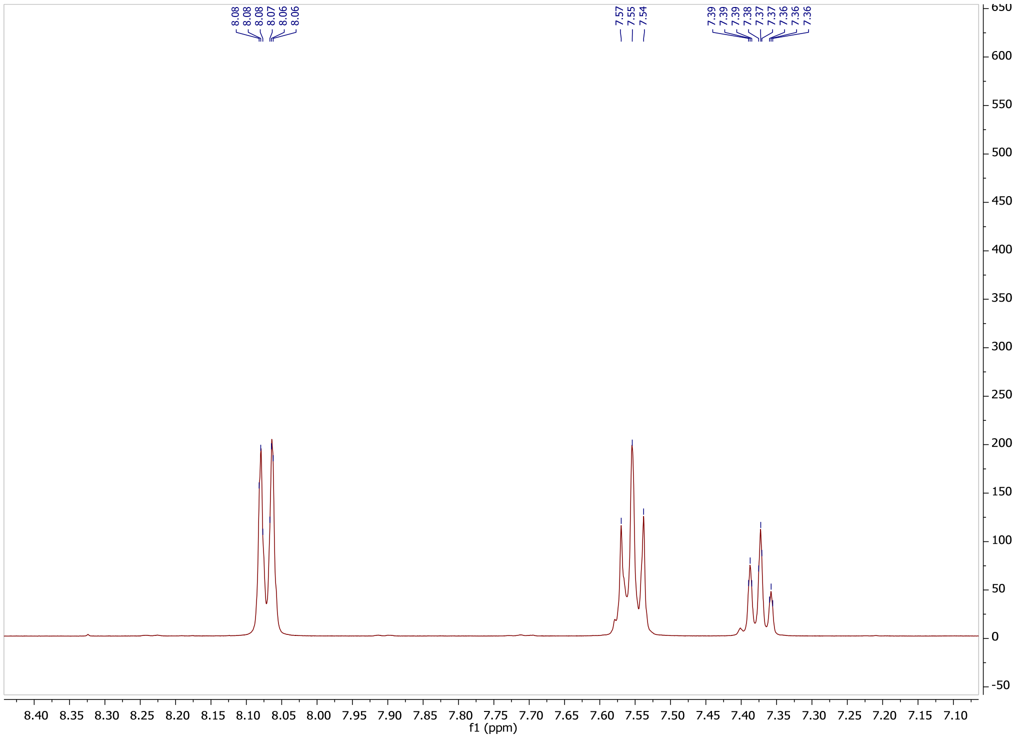

gHSQC-experiment

gHMBC-experiment

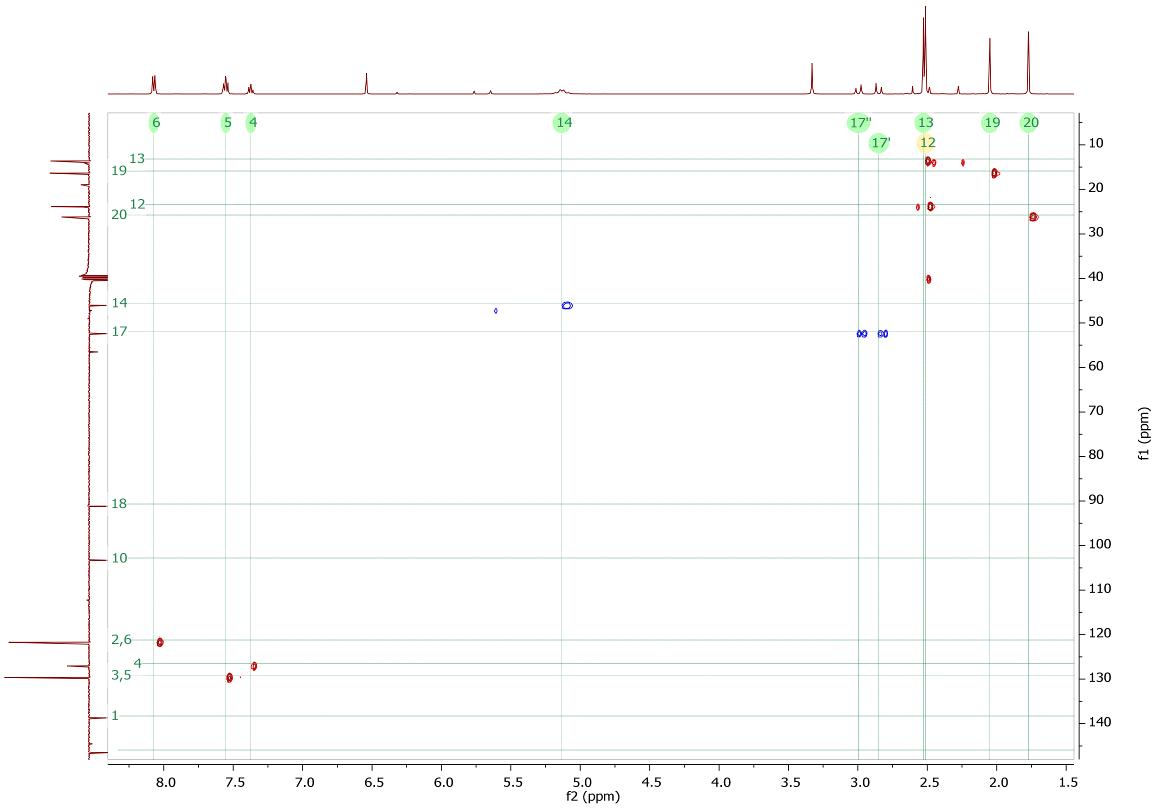

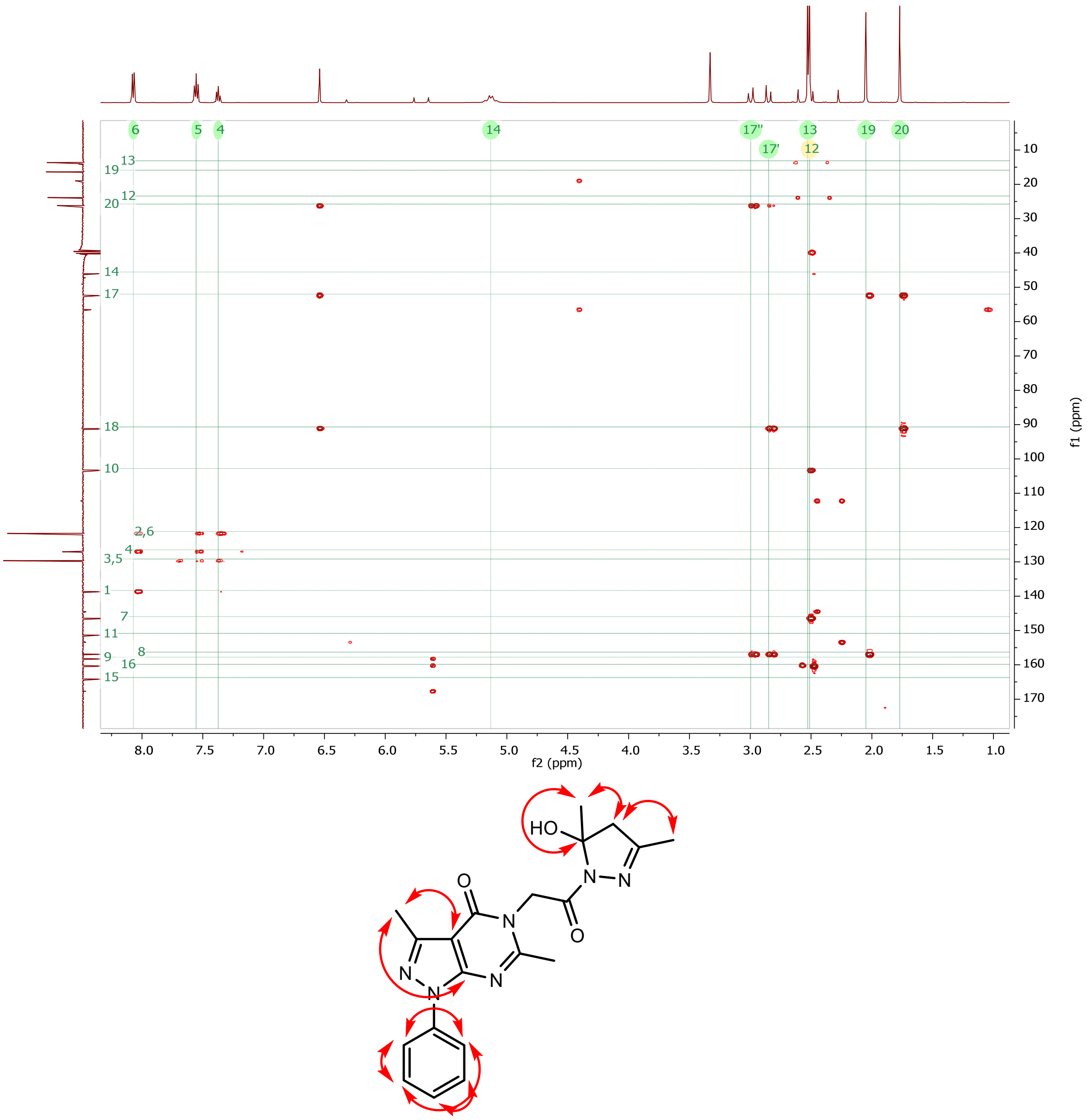
6
5
4
14
17''
17'
13
12
19
20
f2 (ppm)
f1 (ppm)
2,6
3,5
1
7
11
9
8
16
15
18
10
HO
N
O

COSY-experiment

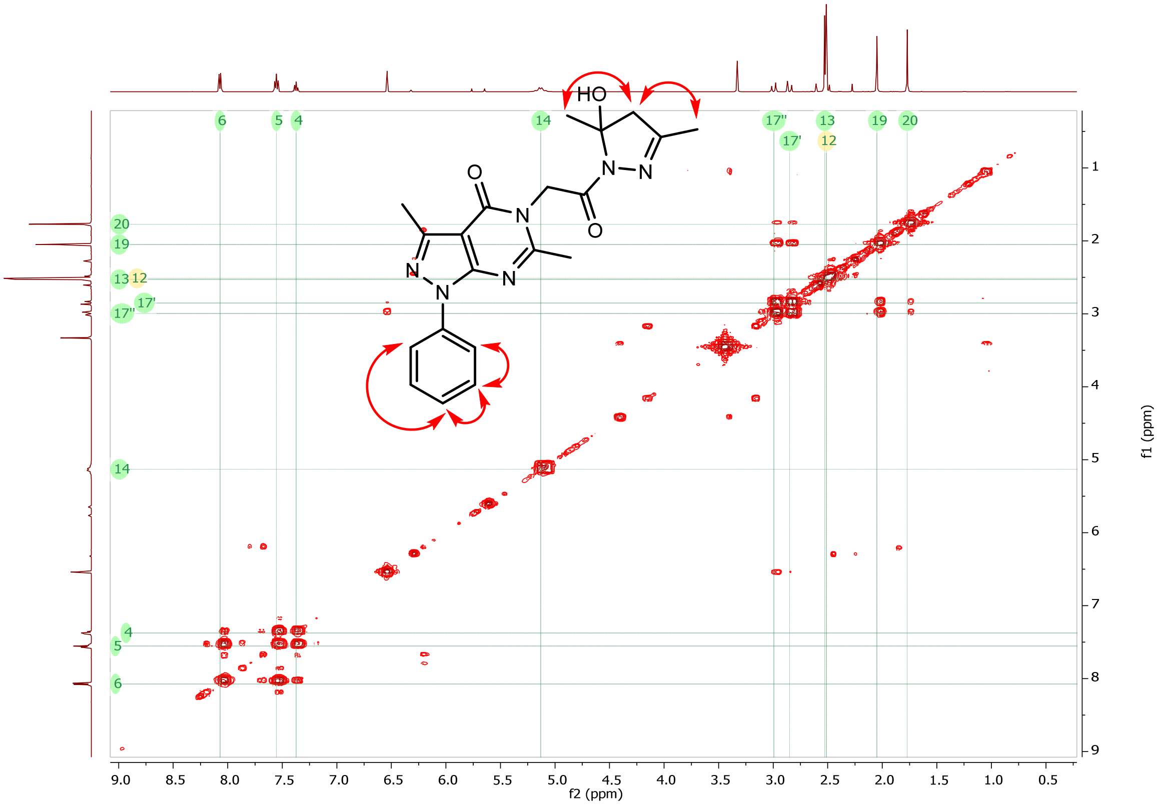

NOESY-experiment

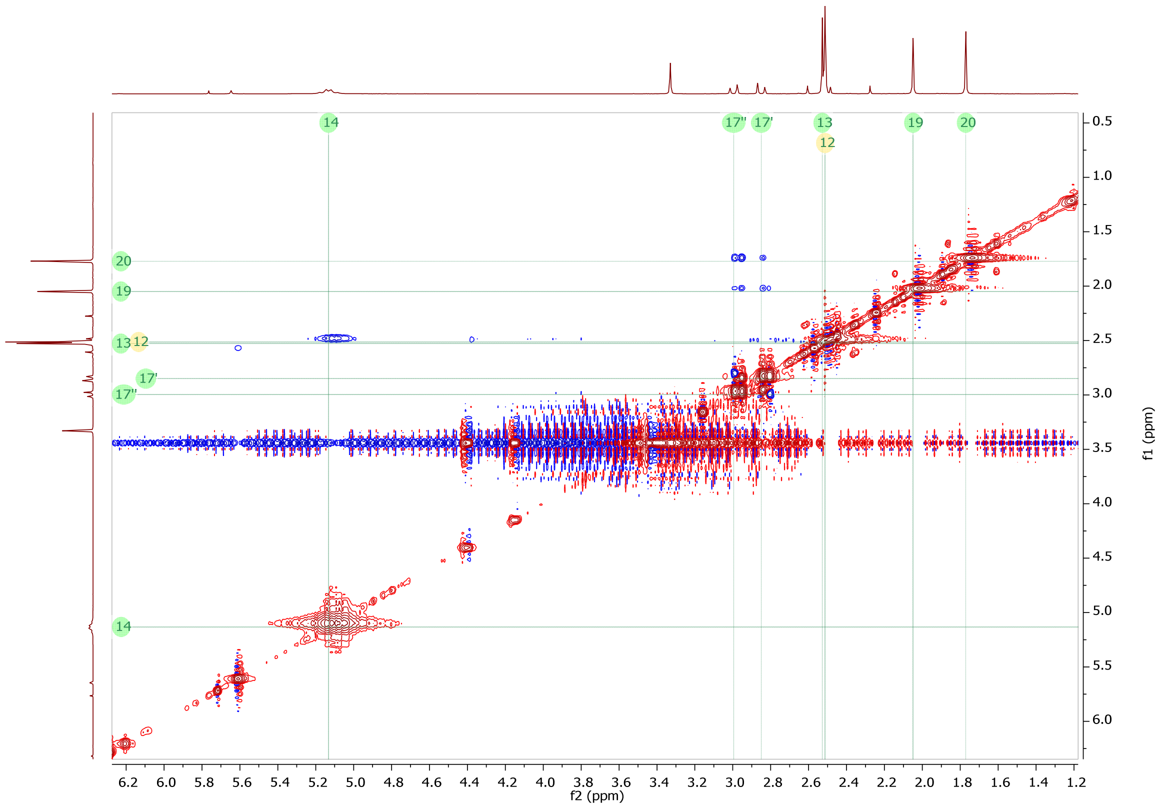

^{13}C-NMR (126 MHz, d-DMSO)

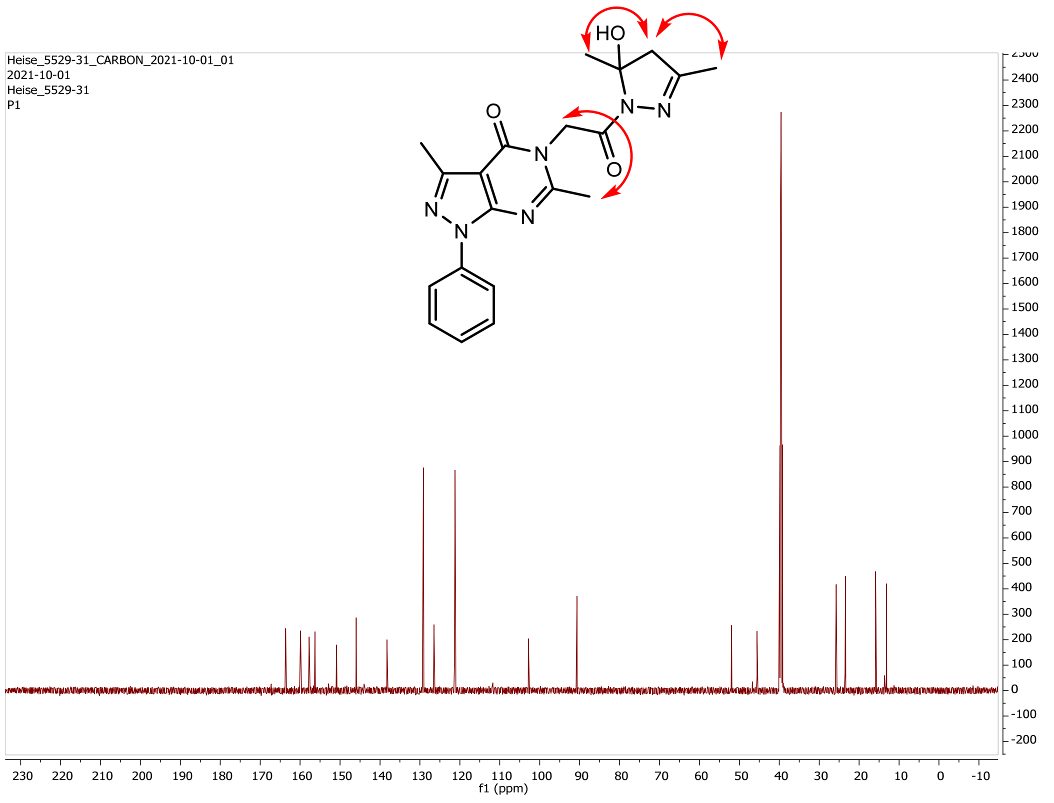

APT- experiment

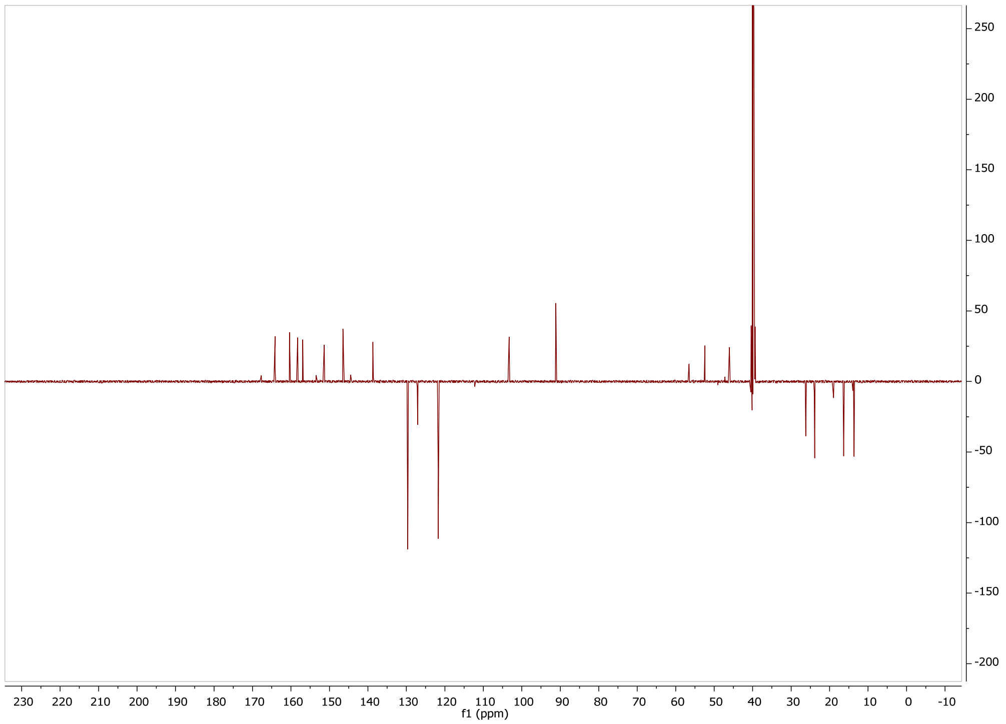

Compound (6)

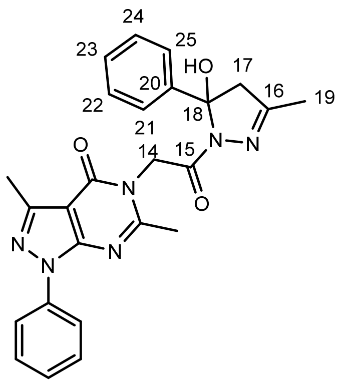

NMR Spectra of **6**

^{1}H-NMR (500 MHz, d-DMSO)

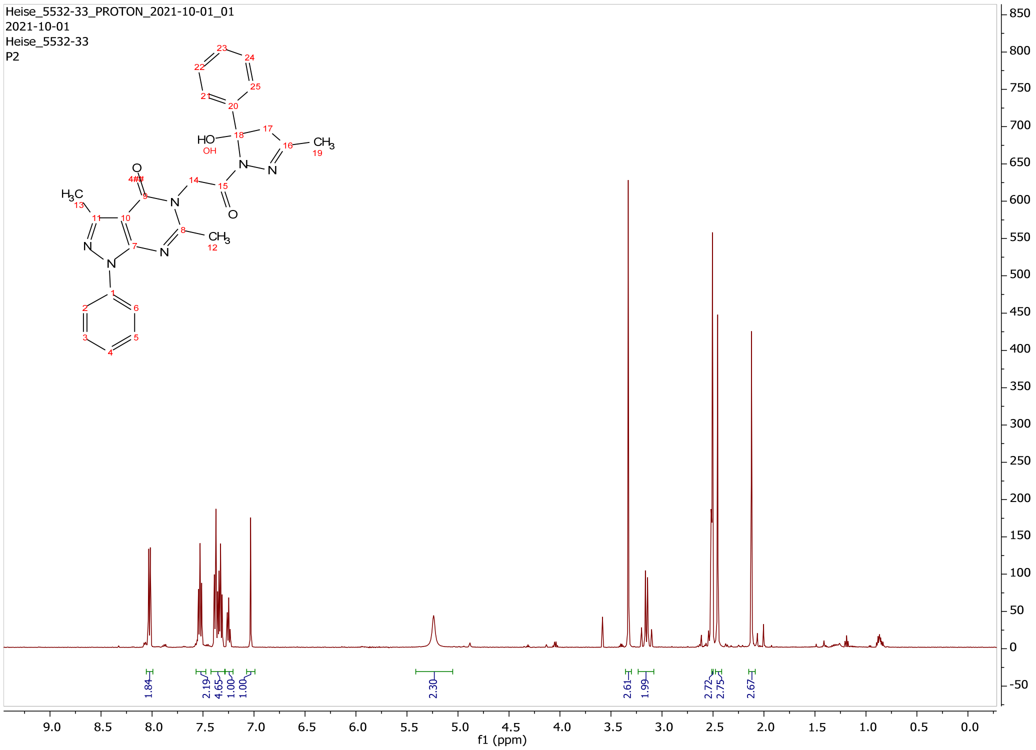

^{13}C-NMR (126 MHz, d-DMSO)

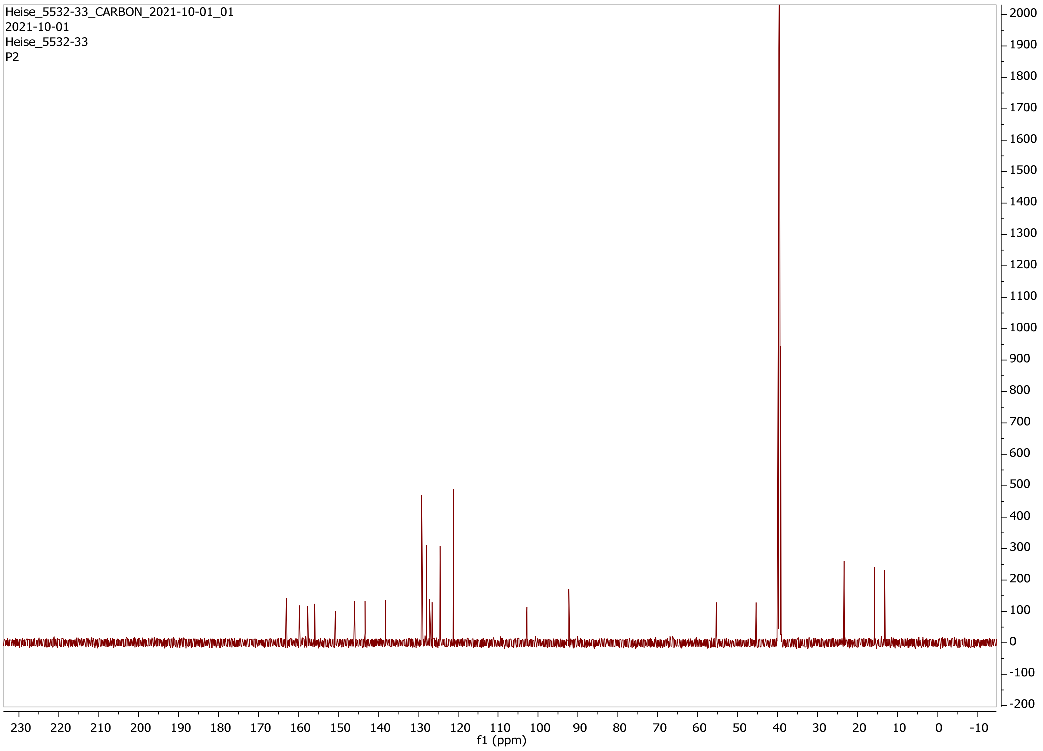

Compound (7)

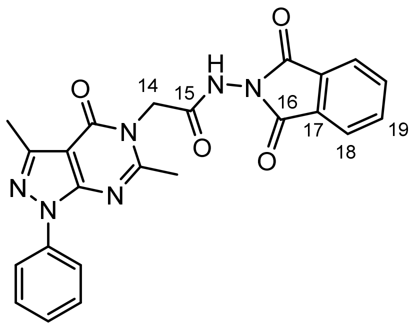

^{1}H-NMR (500 MHz, d-DMSO)

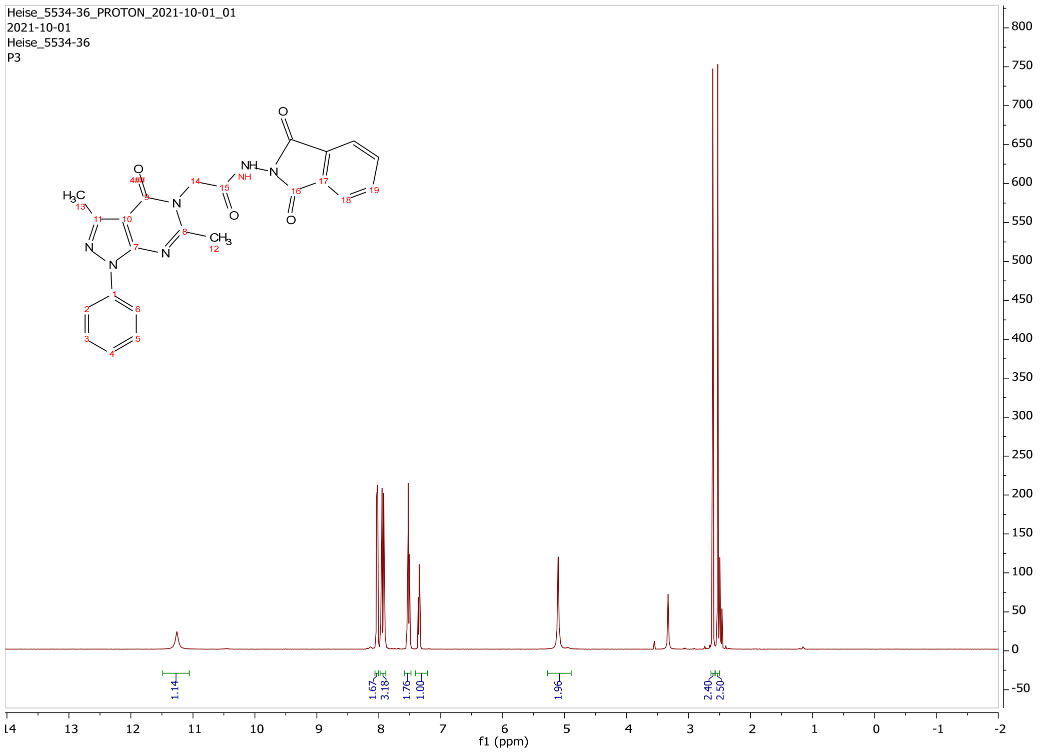

Expansions of the ^{1}H NMR

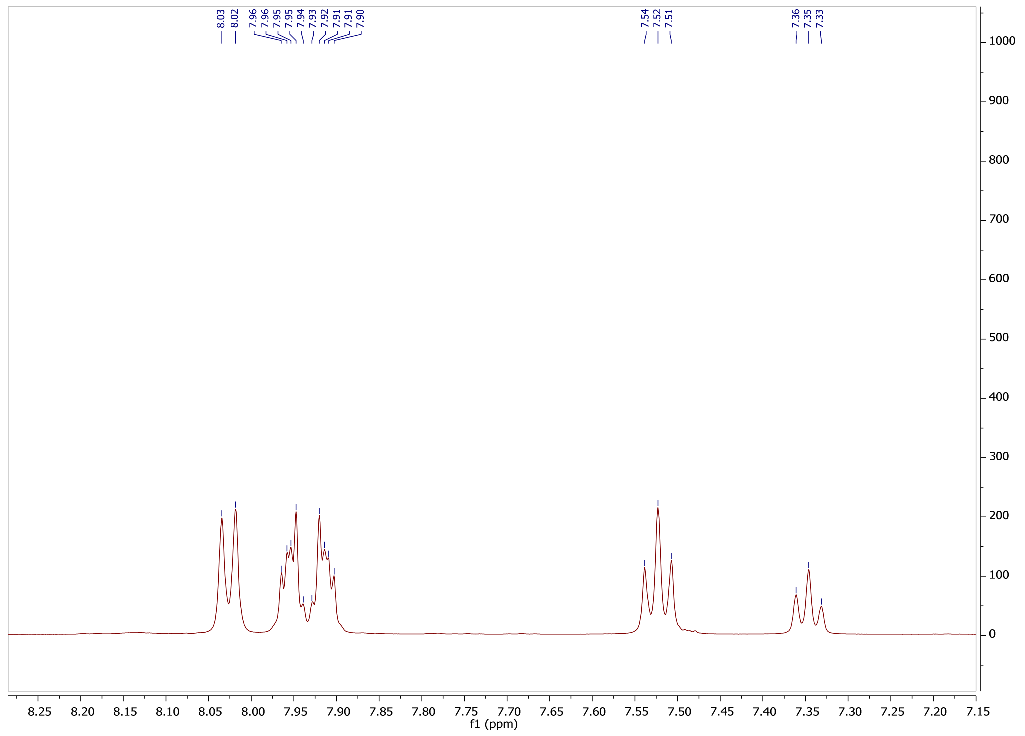

gHSQC-experiment

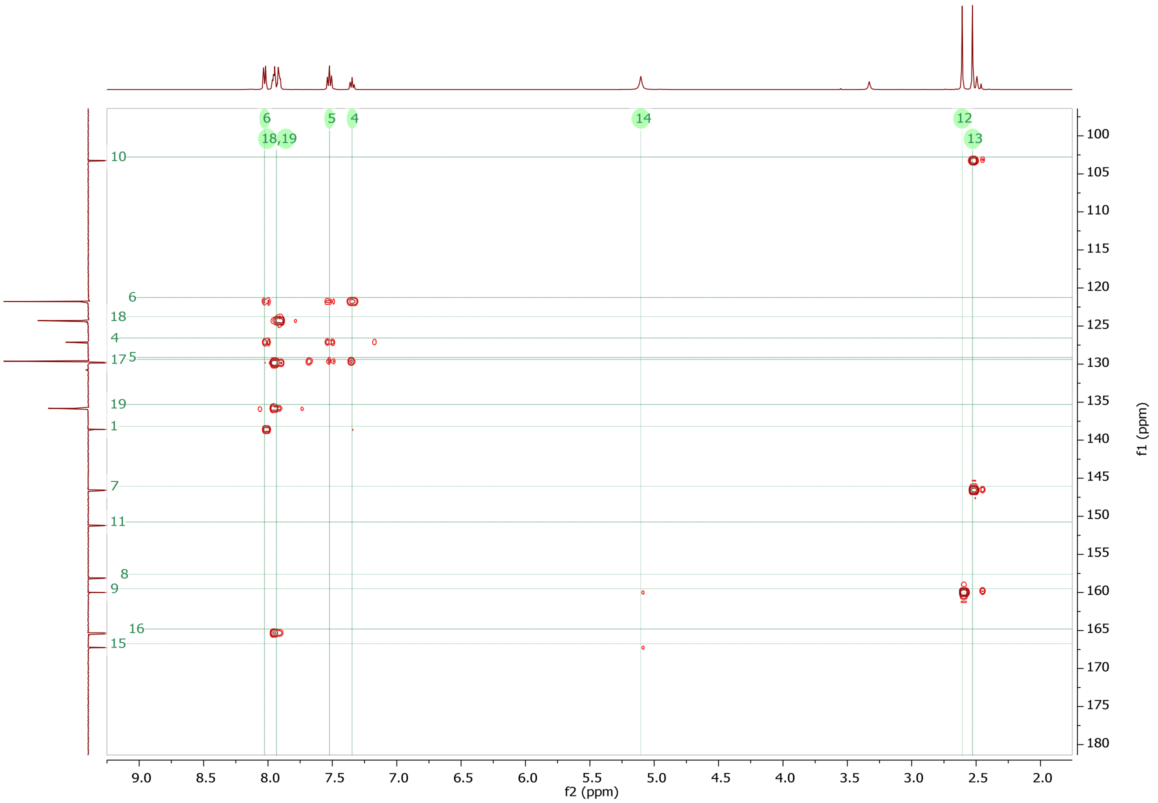

gHMBC-experiment

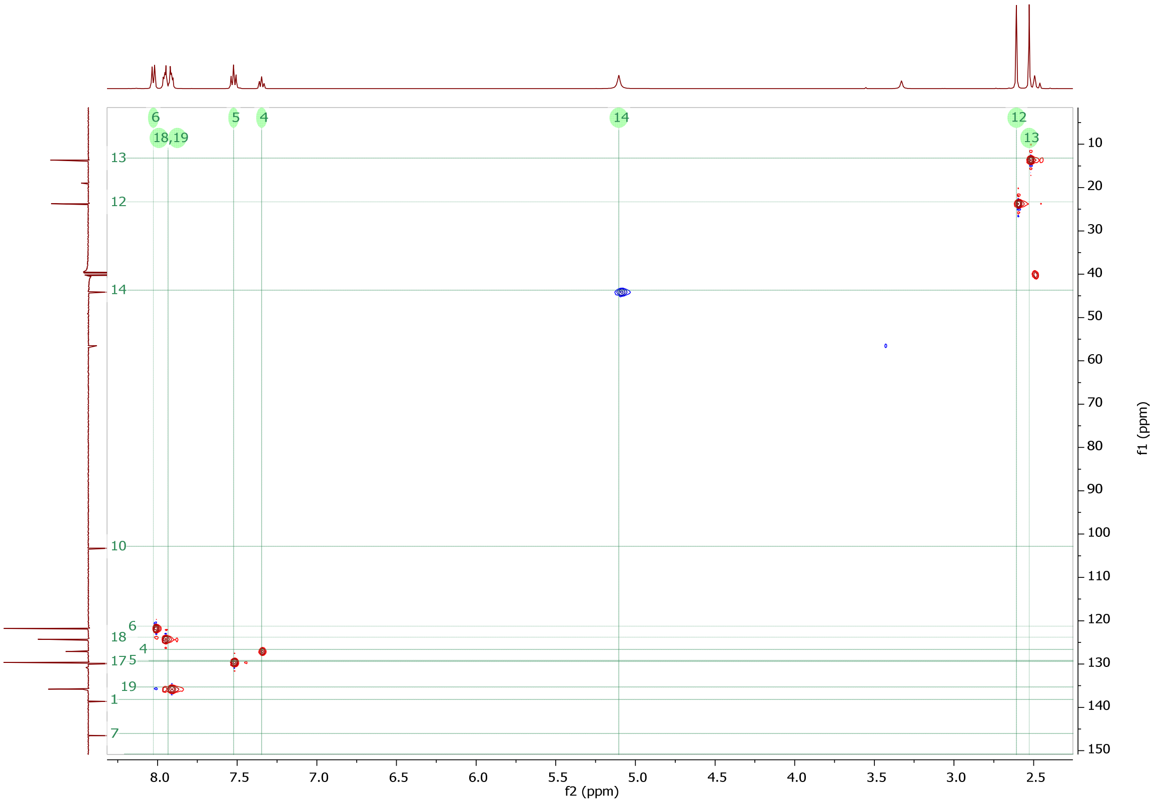

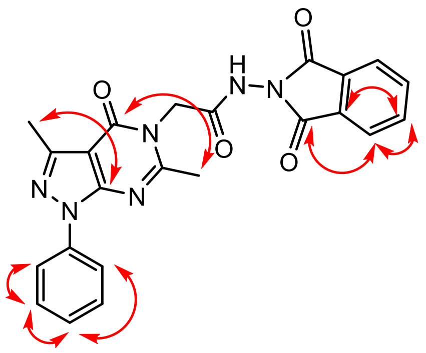

COSY-Experiment

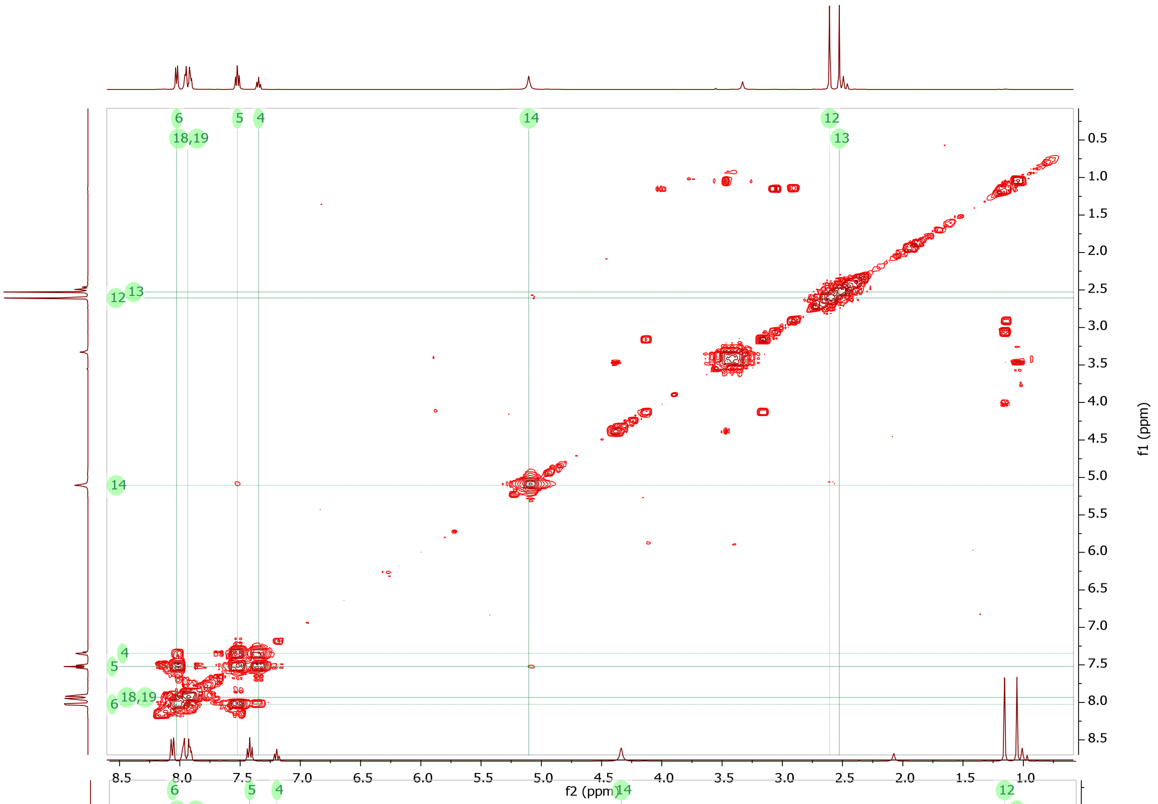

NOESY-

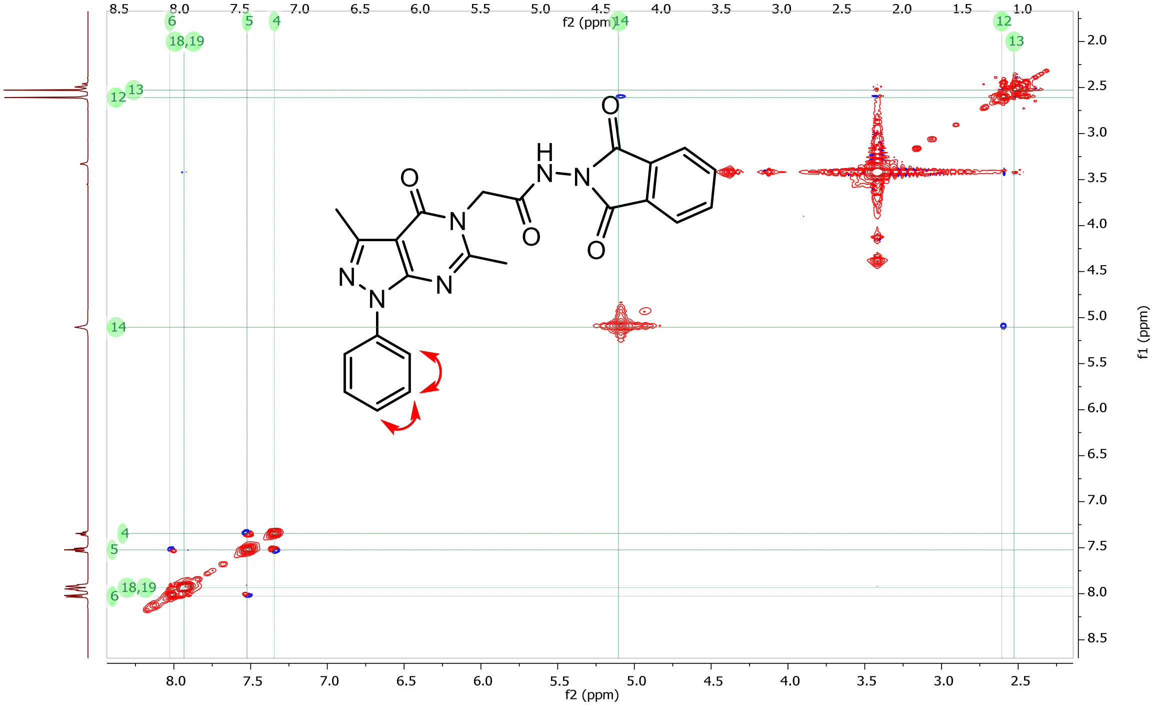

experiment

^{13}C-NMR (126 MHz, d-DMSO)

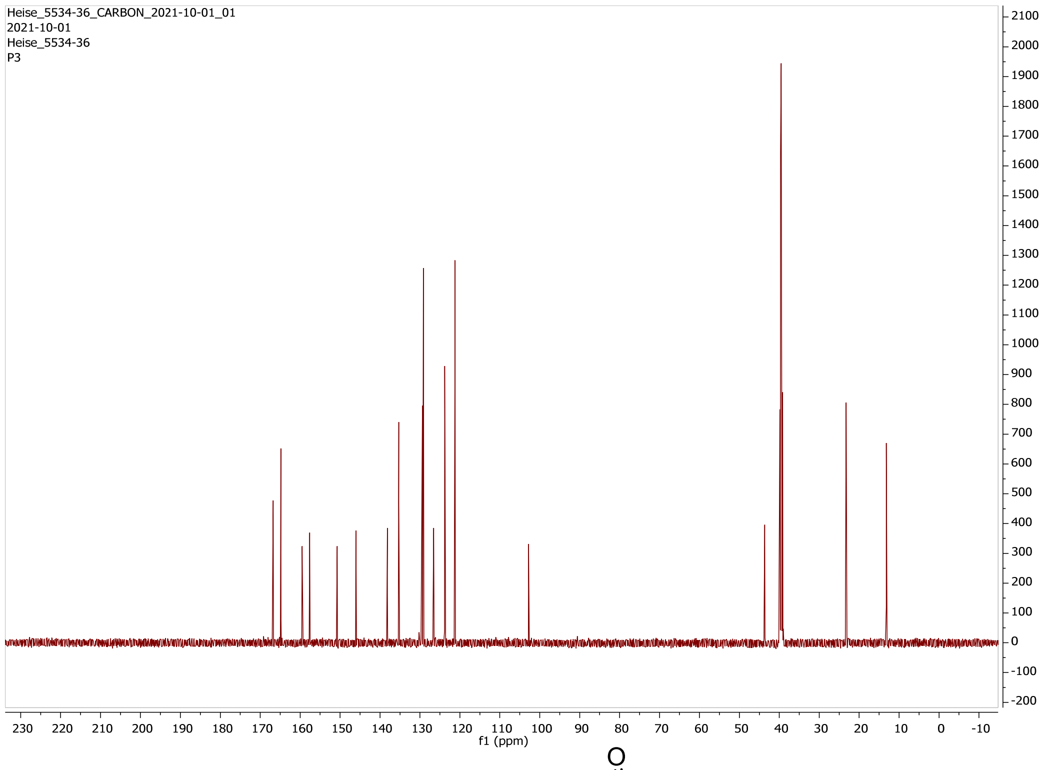

APT-experiment

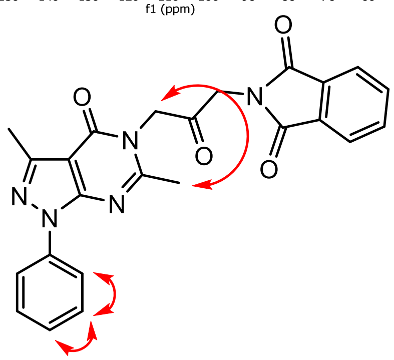

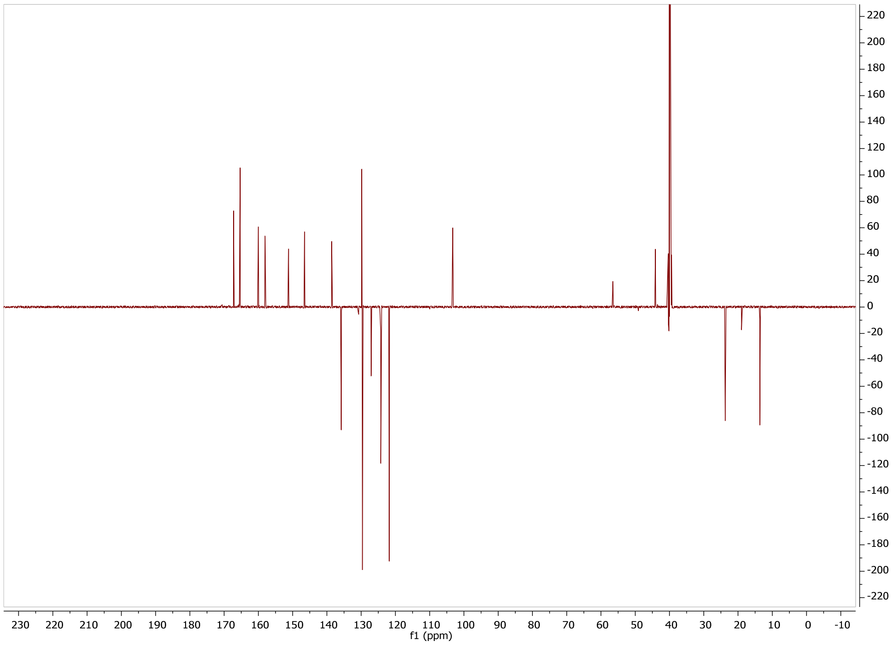

Compound (8)

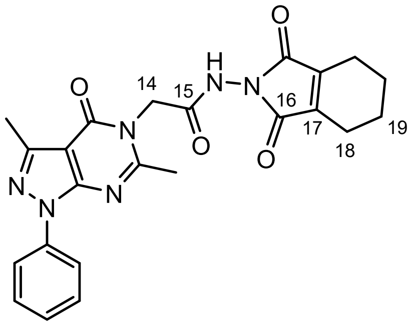

NMR Spectra of **8**

^{1}H-NMR (500 MHz, d-DMSO)

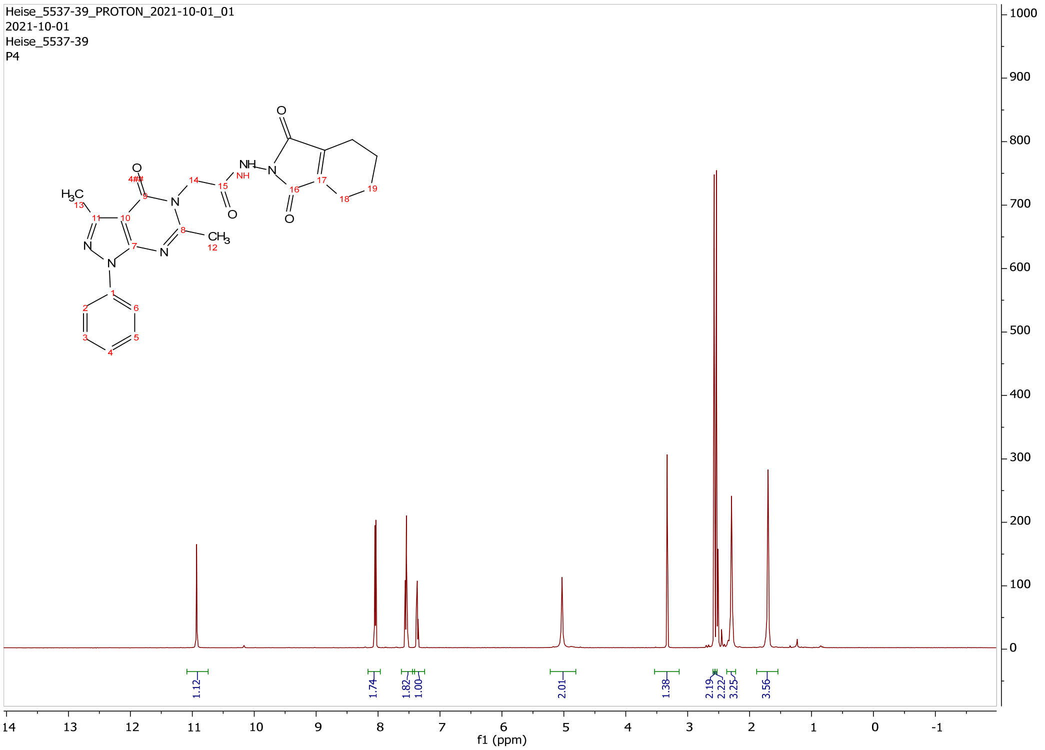

^{13}C-NMR (126 MHz, d-DMSO)

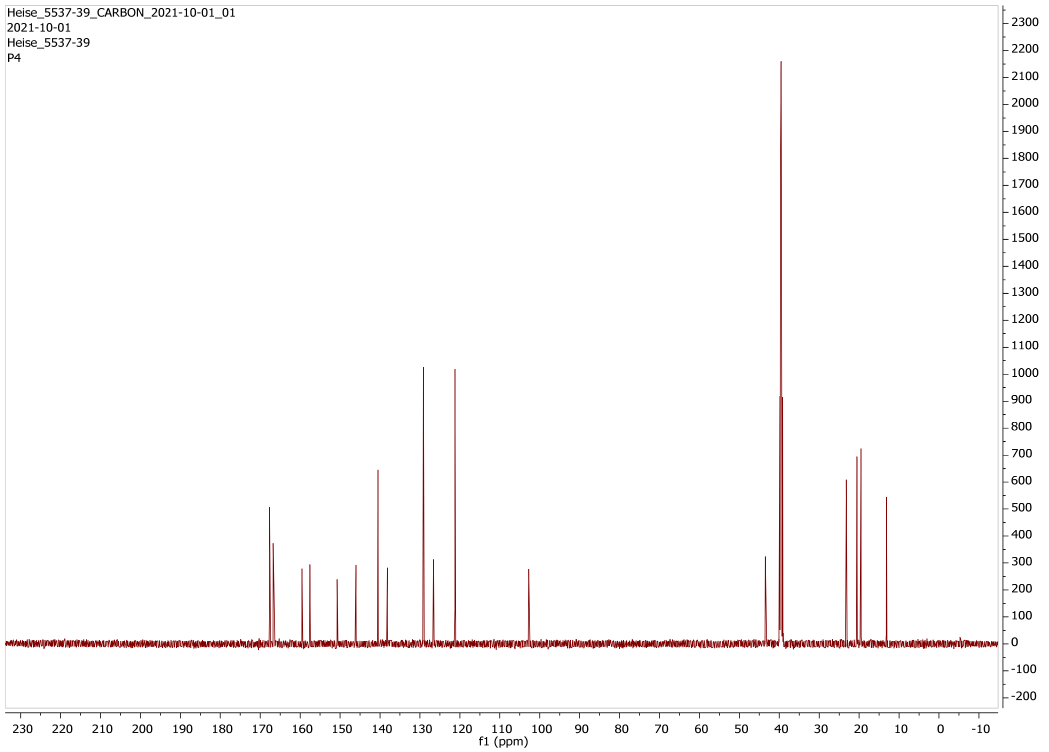

Compound (9)

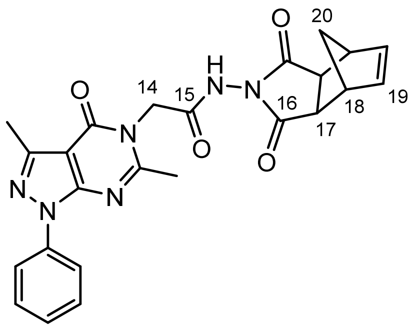

NMR Spectra of **9**

^{1}H-NMR (500 MHz, d-DMSO)

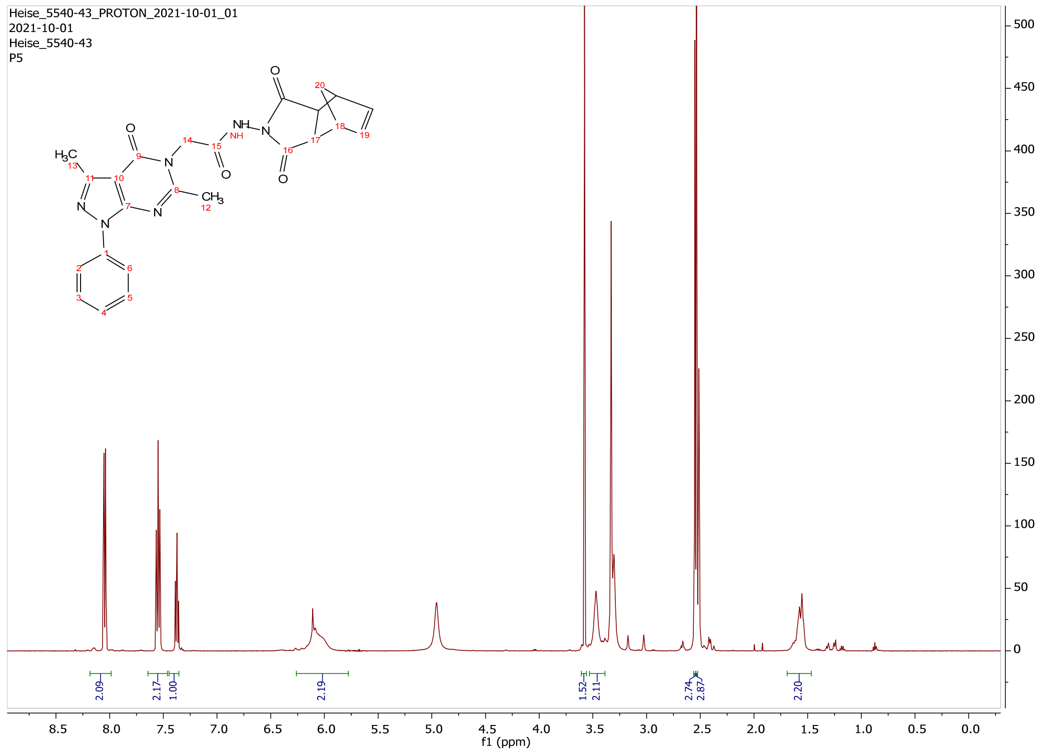

^{13}C-NMR (126 MHz, d-DMSO)

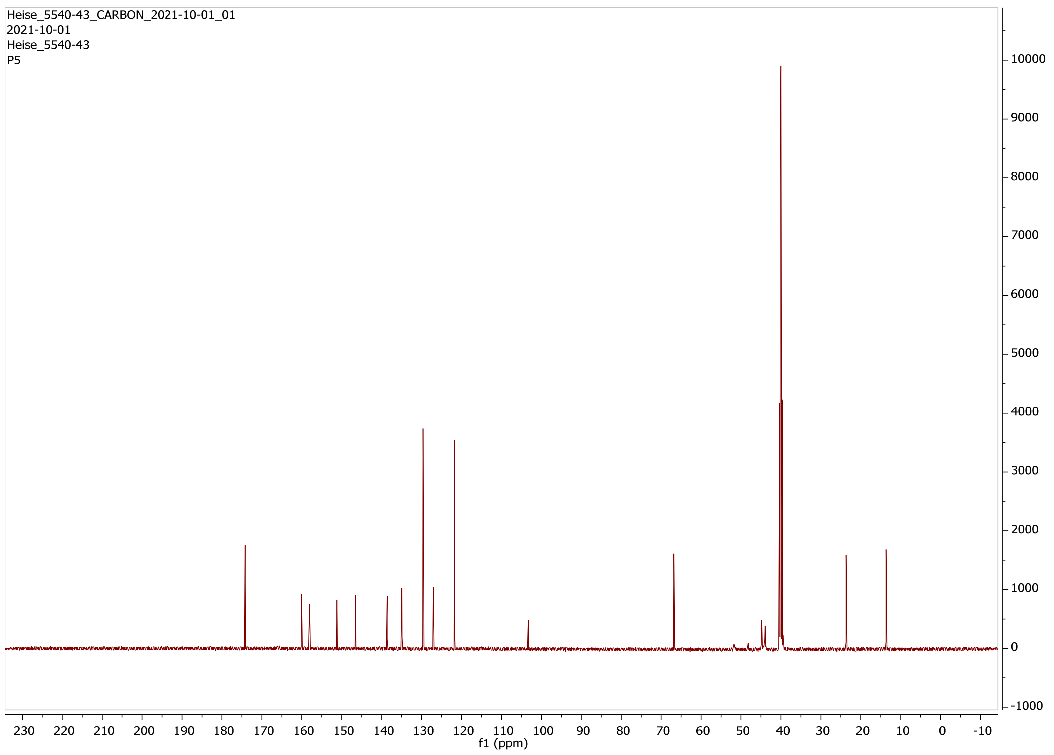

Compound (10)

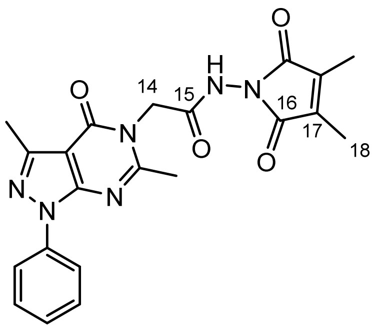

NMR Spectra of **10**

^{1}H-NMR (500 MHz, d-DMSO)

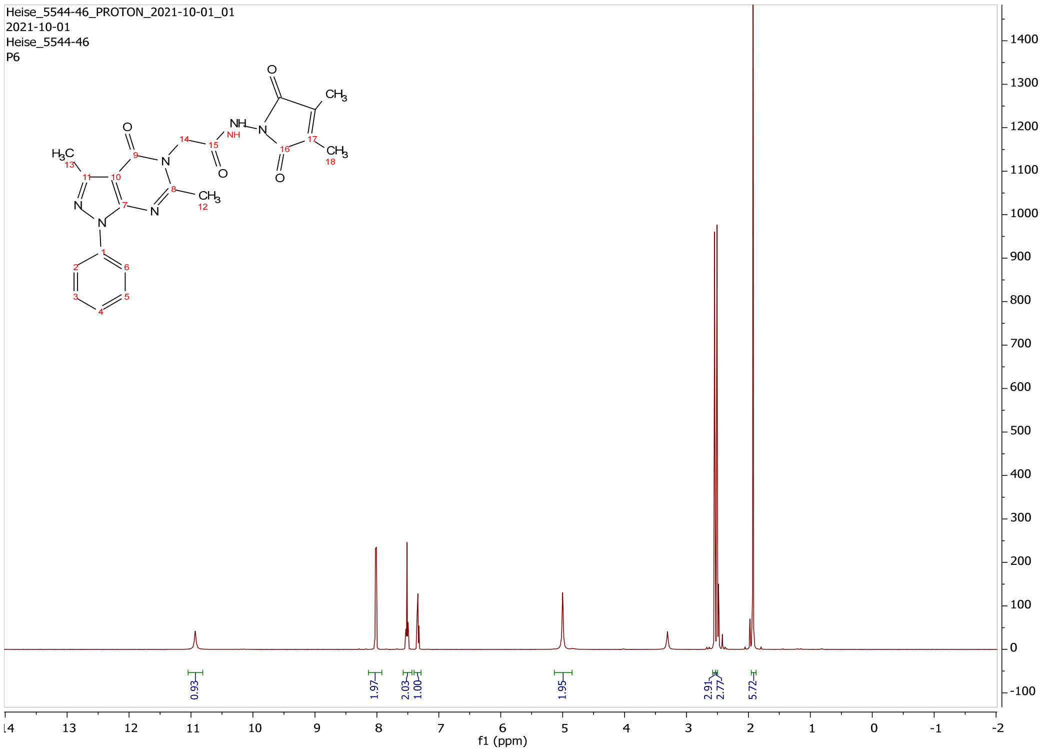

^{13}C-NMR (126 MHz, d-DMSO)

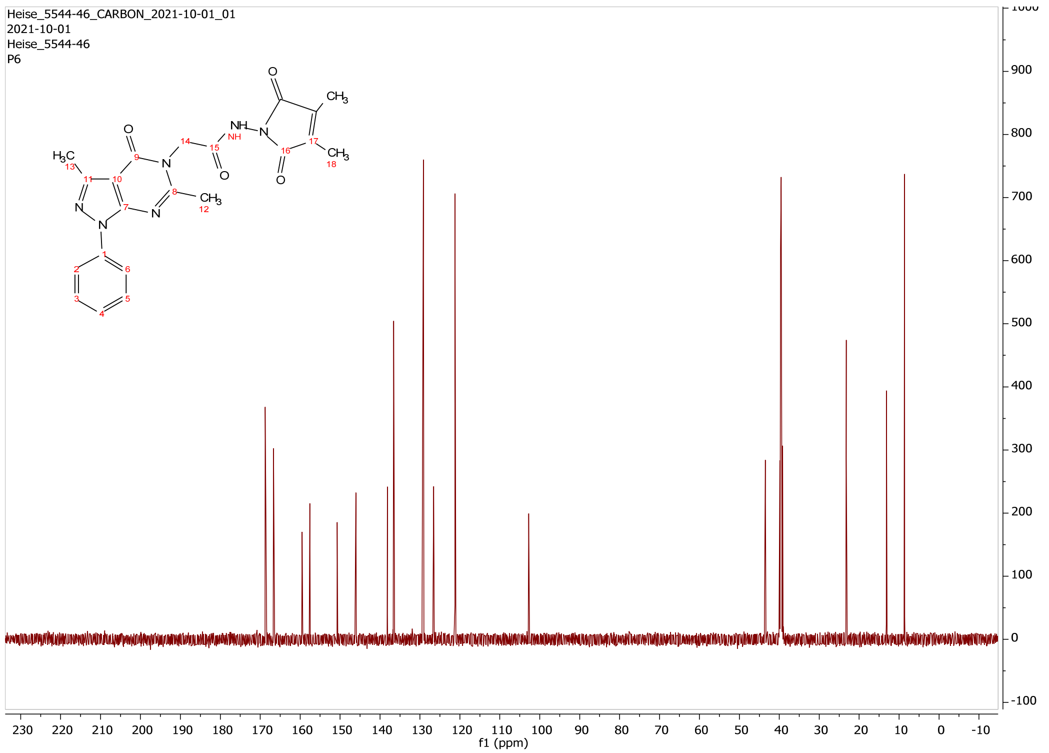

Compound (11)

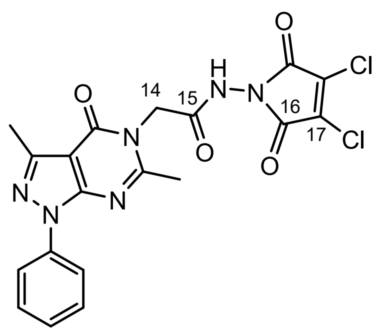

NMR Spectra of **11**

^{1}H-NMR (300 MHz, d-DMSO)

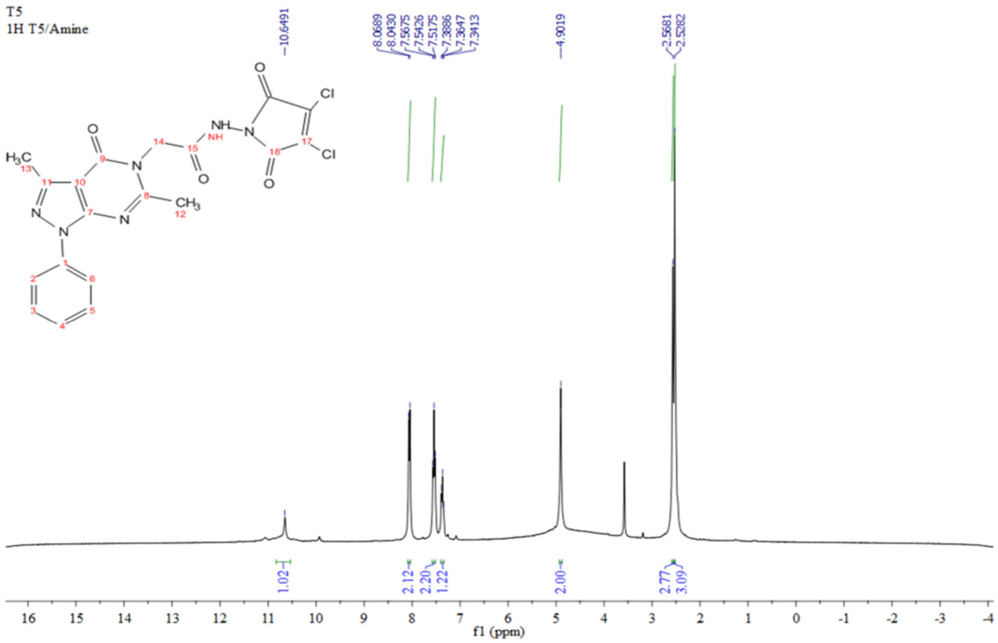

^{13}C-NMR (126 MHz, d-DMSO)

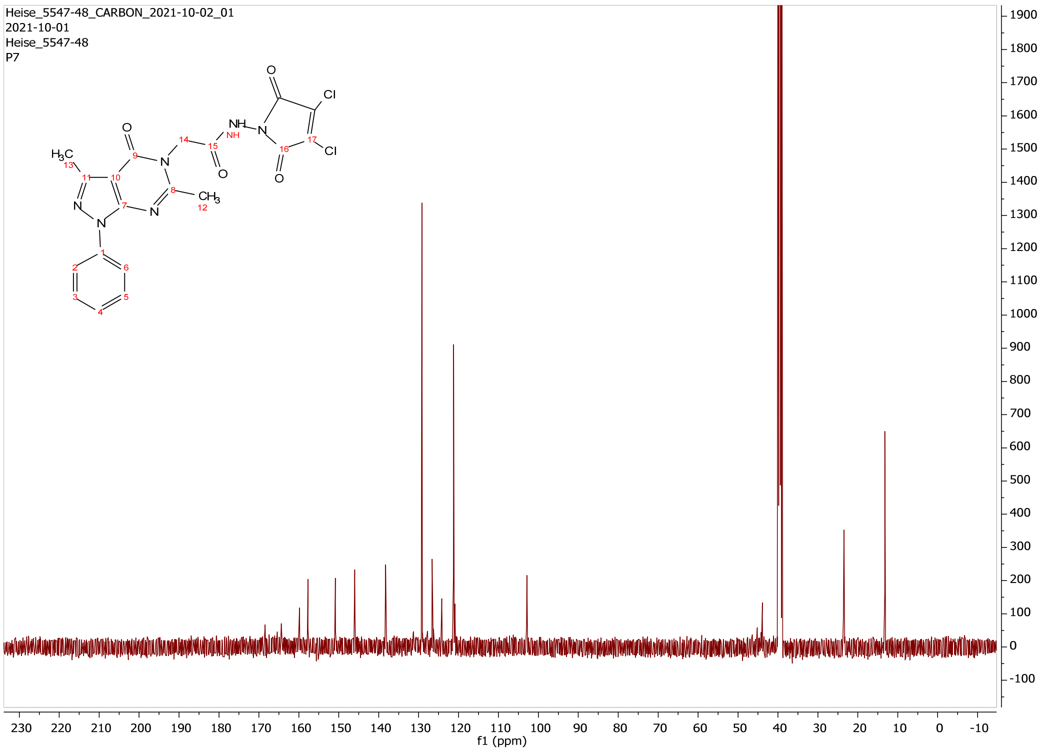

Compound (12)

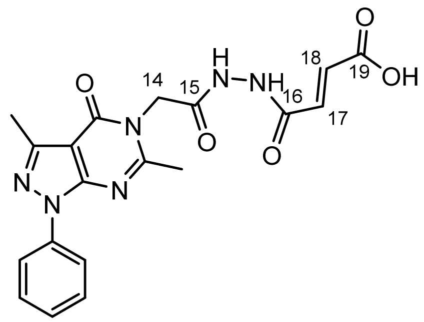

NMR Spectra of **12**

^{1}H-NMR (500 MHz, d-DMSO)

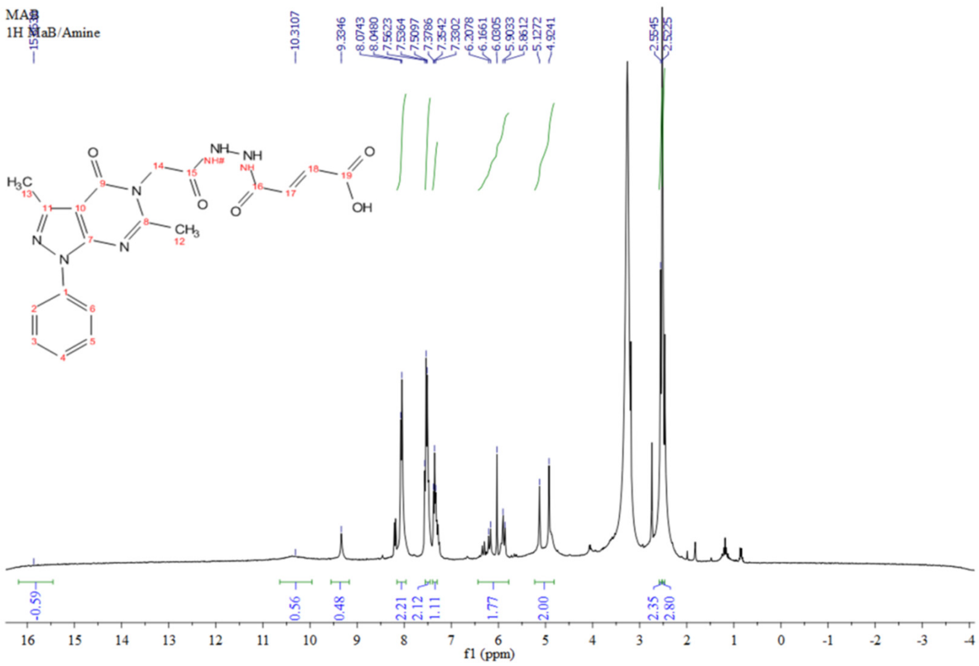

^{13}C-NMR (126 MHz, d-DMSO)

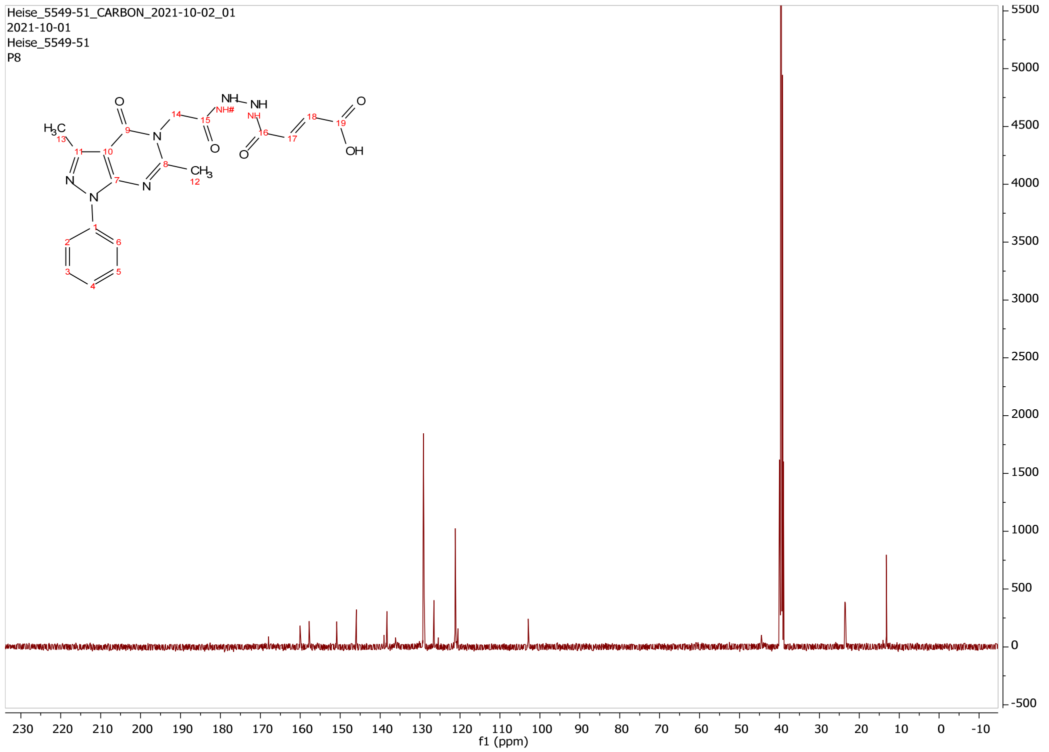

Compound (13)

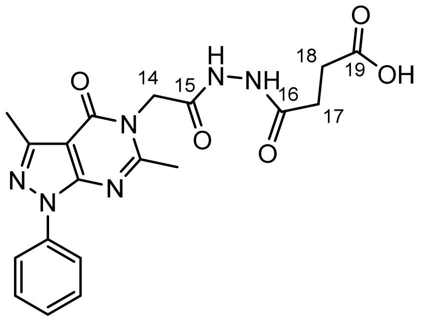

NMR Spectra of **13**

^{1}H-NMR (500 MHz, d-DMSO)

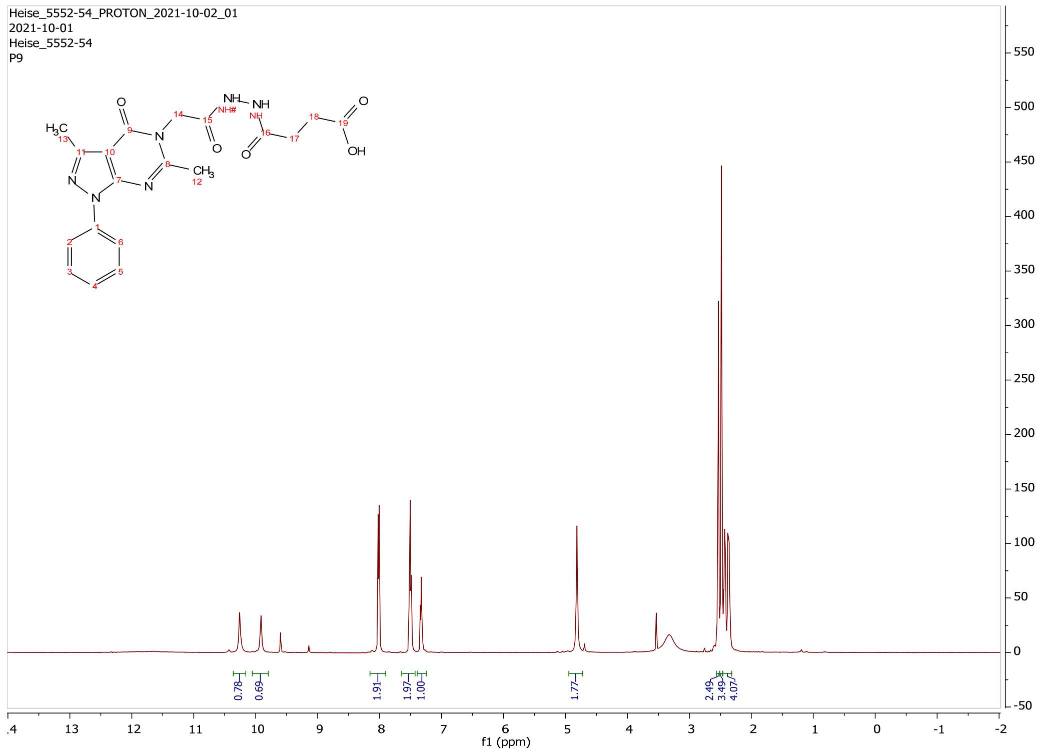

^{13}C-NMR (126 MHz, d-DMSO)

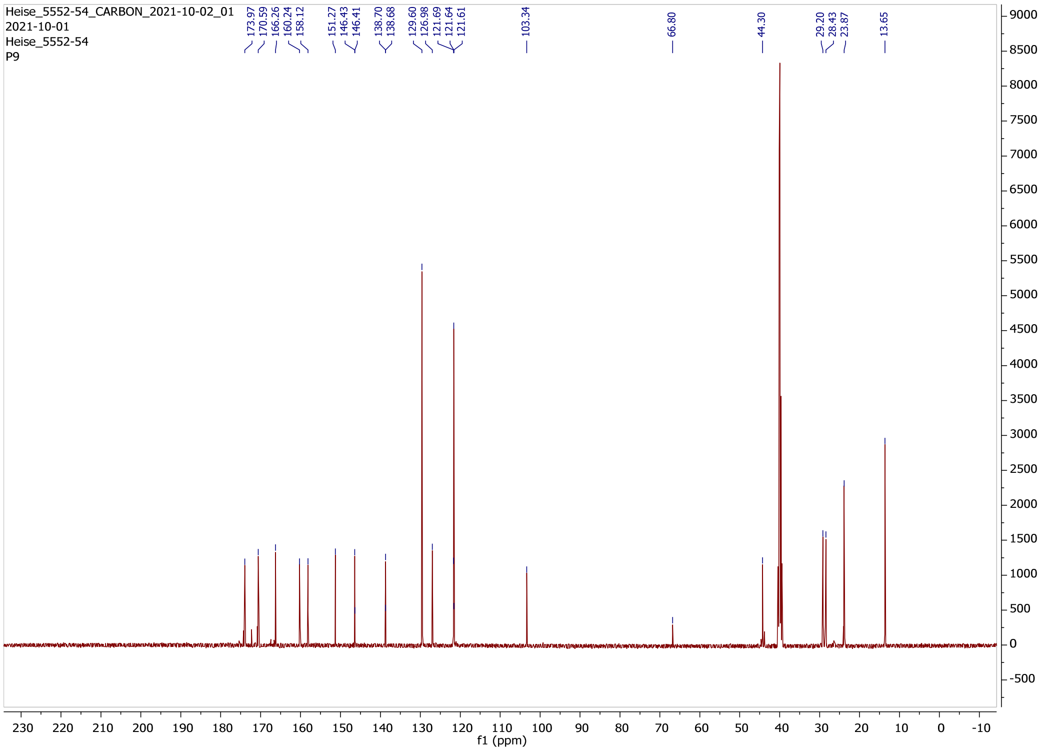

Supplement: Supplementary file 1 [file molecules-27-05303-s001.zip › molecules-1867970-supplementary.pdf]
